# Floral scent profiling and candidate gene discovery in *Hesperis oreophila*

**DOI:** 10.3389/fpls.2026.1922330

**Published:** 2026-08-03

**Authors:** Jinge Xu, Xinran Zhao, Wenxiao Liu, Lingjie Xu, Jinghao Tang, Qingjuan Nie, Shun Cheng, Yanhui Li

**Affiliations:** 1 College of Landscape Architecture and Tourism, Hebei Agricultural University, Baoding, China; 2 Saihanba Mechanical Forest Farm, Chengde, Hebei, China

**Keywords:** differentially expressed genes, floral scent, *Hesperis oreophila*, metabolome, terpenoid biosynthesis, transcriptome

## Abstract

*Hesperis oreophila* is a wild cruciferous aromatic plant endemic to North China, valued for both ornamental appeal and fragrance development. However, the molecular regulatory mechanisms underlying floral scent formation in *Hesperis* have not been systematically elucidated. In this study, HS-SPME-GC-MS-based VOC profiling was performed across four floral developmental stages, including small bud (P1), large bud (P2), initial bloom (P3), and full bloom (P4), whereas transcriptome sequencing was conducted for three scent-transition stages, P2, P3, and P4. A total of 1,211 volatile organic compounds (VOCs) were detected and putatively annotated. Terpenoids were the predominant scent constituents at the initial and full bloom stages, reaching 166.25 μg·g^−1^ at full bloom. Relative odor activity value (rOAV) analysis suggested that citronellol, myrcene, and limonene may be important putative aroma-contributing compounds, and the initial bloom stage was revealed as a major developmental transition associated with increased VOC accumulation. Genes involved in the MVA pathway showed relatively higher expression at the large bud stage, whereas several MEP pathway genes and terpene synthase genes were upregulated at the initial bloom stage, suggesting stage-specific transcriptional patterns associated with terpenoid accumulation. Concurrently, several phenylpropanoid-related genes also showed elevated expression at P3, suggesting coordinated developmental changes in scent-related pathways. A gene–metabolite correlation network identified *MVK*, *DXS*, and *TPS22* as candidate genes associated with terpenoid VOC accumulation. Among them, *TPS22* was significantly positively correlated with multiple characteristic terpenoids and may represent a promising candidate gene associated with terpenoid compositional variation. This study provides initial molecular and metabolic insights into floral scent formation in *H. oreophila*, providing a theoretical foundation and candidate gene resources for germplasm conservation, molecular breeding, and industrial development.

## 1 Introduction

Floral scent, together with flower color and shape, is a key ornamental trait that determines the aesthetic and economic value of flowering plants (Ramya et al., 2020). As low-molecular-weight volatile secondary metabolites released by plants, floral scents not only act as key chemical signals mediating plant–pollinator interactions and defense against biotic stress (Xiao et al., 2013), but also significantly enhance the therapeutic value and recreational experience of garden landscape (Zhang et al., 2016; Sánchez-Vidaña et al., 2017; Lindgren et al., 2019); moreover, their extracts possess high commercial development potential in cosmetics, food flavoring, and pharmaceutical industries (Yuan et al., 2020; Breuninger et al., 2026). Research on floral traits has primarily centered on visual attributes such as flower color and shape (Delle-Vedove et al., 2017); thus far, more than 2,000 volatile compounds have been identified from over 60 plant families (Knudsen et al., 2006; Farré-Armengol et al., 2020); however, the elucidation of the molecular mechanisms underlying floral scent formation has lagged considerably, and studies on wild native aromatic plants remain particularly scarce.

Plant floral scent compounds are primarily classified into three major groups: terpenoids, phenylpropanoids/benzenoids, and fatty acid derivatives (Muhammad et al., 2022). As the core volatile constituents of floral scent, terpenoids are biosynthesized through the cooperative regulation of two evolutionarily conserved and subcellularly compartmentalized metabolic pathways. The plastid-localized 2-C-methyl-D-erythritol 4-phosphate (MEP) pathway generates the monoterpene precursor geranyl diphosphate (GPP) through a cascade of key rate-limiting enzymes, thereby predominantly supplying the carbon skeletons for monoterpene aromas. The mevalonate (MVA) pathway, localized in the cytosol and endoplasmic reticulum, employs 3-hydroxy-3-methylglutaryl-CoA reductase (HMGR) as the core rate-limiting node to synthesize the sesquiterpene precursor farnesyl diphosphate (FPP), responsible for the precursor supply of sesquiterpenes. Rather than functioning independently, these two pathways undergo precursor exchange and coordinated regulation, and ultimately, through the functional diversification of the terpene synthase (*TPS*) family, structurally diverse terpenoid scent compounds are produced (Tholl, 2015; Pu et al., 2021; Chen and Zhang, 2020). The terpene synthase (*TPS*) gene family represents the core catalytic component underlying the structural and functional diversity of terpenoid scent compounds. Its distinct subfamilies (e.g., *TPS-a*, *TPS-b*, *TPS-g*) exhibit pronounced substrate preferences and subcellular functional differentiation, constituting the key molecular basis for interspecific variation in floral terpenoid composition (Jia et al., 2022). Functional validation of key *TPS* genes has been accomplished in multiple plant species: in *Lilium* ‘Siberia’, *LoTPS2* primarily catalyzes the synthesis of (E,E)-α-farnesene (Abbas et al., 2020); in wild *Freesia* species, the biosynthesis of β-caryophyllene and nerolidol is specifically regulated by *TPS10* and *TPS14*, respectively (Bao et al., 2023; Gao et al., 2018); a systematic analysis of wild *Paeonia* groups and cultivars by Li et al. (2023) suggested that *PdTPS1* and *PdTPS4* catalyze the conversion of the precursor GPP to linalool, serving as the core genes determining species- and cultivar-specific linalool emission. Phenylpropanoids, using phenylalanine as a substrate, form volatile benzenoid compounds through the action of key enzymes such as phenylalanine ammonia-lyase (PAL); fatty acid derivatives are mainly derived from C18 fatty acids via the lipoxygenase (LOX) pathway (Wasternack and Feussner, 2018). Most volatile compounds are synthesized and emitted from petal epidermal cells (Dudareva et al., 2004), and their metabolic processes are hierarchically regulated by structural genes and transcription factors such as *MYB* and *bHLH* (Yang et al., 2022; Feng et al., 2023; Guo et al., 2023). Although the floral scent pathways in model plants and horticultural crops have been extensively elucidated, systematic studies on the metabolic networks of floral scent in wild flowers remain very limited.

In recent years, the construction of near-natural landscapes has driven the development of wild flower resources, and native aromatic plants have drawn considerable attention owing to their strong adaptability and distinctive fragrance (Gao, 2017; Bai, 2024). *Hesperis oreophila*, a perennial herb of the genus *Hesperis* (Brassicaceae), is a key protected wild plant in Hebei Province. It produces large racemes with vibrant flower colors and emits a rich fragrance that is especially pronounced at night, thus holding great promise for landscape application and fragrance development (Majetic et al., 2007). To date, research on the genus *Hesperis* has primarily focused on species taxonomy, phylogeny, morphological characteristics, and preliminary detection of volatile components (Hwang and Lauenroth, 2010; Kirimer et al., 2010; Hloušková et al., 2019; Eslami-Farouji et al., 2021). Studies on *H. oreophila* in China have been limited to germplasm surveys, introduction and domestication, photosynthetic physiology, and propagation techniques (Li et al., 2024; Liu et al., 2019; Dong et al., 2022, 2023). The dynamic changes of its floral scent volatiles across flowering stages, the putative aroma-contributing compounds, the core biosynthetic pathways, and the molecular regulatory mechanisms have not been reported thus far, severely restricting the in-depth utilization and targeted genetic improvement of this valuable wild germplasm.

Previous studies on *Hesperis* species have mainly relied on taxonomic, physiological, or single-platform volatile analyses, which can describe species characteristics or partial scent composition but cannot explain how scent-related metabolites are developmentally coordinated with gene expression. In particular, single-component volatile detection is insufficient to resolve the temporal activation of terpenoid, phenylpropanoid/benzenoid, and fatty-acid-derived pathways, while existing physiological studies of *H. oreophila* do not reveal the molecular basis of scent formation. Therefore, an integrated volatile metabolome–transcriptome strategy is required to simultaneously characterize dynamic VOC accumulation, identify putative aroma-contributing compounds, and mine candidate genes associated with floral scent biosynthesis in this non-model wild Brassicaceae species.

The combined analysis of GC–MS-based volatile metabolomics and transcriptomics has become a mainstream and efficient technical strategy for elucidating the molecular regulatory mechanisms of plant floral scent (Park et al., 2020). This approach enables the accurate characterization of accumulation patterns of aroma compounds at different flowering stages and the systematic mining of key metabolic pathways, structural genes, and transcriptional regulators. It has been successfully applied to floral scent studies in multiple plant species, including *Prunus mume*, *Magnolia champaca*, *Hydrangea arborescens*, and *Lonicera japonica* (Dhandapani et al., 2017; Li et al., 2022; Wang et al., 2022; Ke et al., 2023), thereby offering a mature and reliable technical pathway for the mechanistic elucidation of non-model wild aromatic plants.

Given this background, the present study used floral organs of *H. oreophila* at four developmental stages—small bud (P1), large bud (P2), initial bloom (P3), and full bloom (P4)—to characterize developmental changes in floral VOCs. Based on the VOC profiles, transcriptome sequencing was further performed for P2, P3, and P4, which represented the transition from the bud stage to active scent emission. The integrated transcriptome–metabolome analysis was therefore restricted to P2–P4, allowing us to systematically investigate the dynamic emission rhythms of scent compounds during flower development, identify the putative aroma-contributing compounds and core biosynthetic pathways, mine the potential key genes regulating floral scent formation, and preliminarily construct the candidate gene–metabolite association network underlying floral scent metabolism. The findings aim to fill the knowledge gap regarding floral scent mechanisms in *H. oreophila* and the genus *Hesperis* at large, and to provide a scientific and theoretical basis for scent trait improvement, molecular breeding, and landscape industrial application of wild aromatic flowers.

## 2 Materials and methods

### 2.1 Plant materials and experimental design

**Figure 1:**
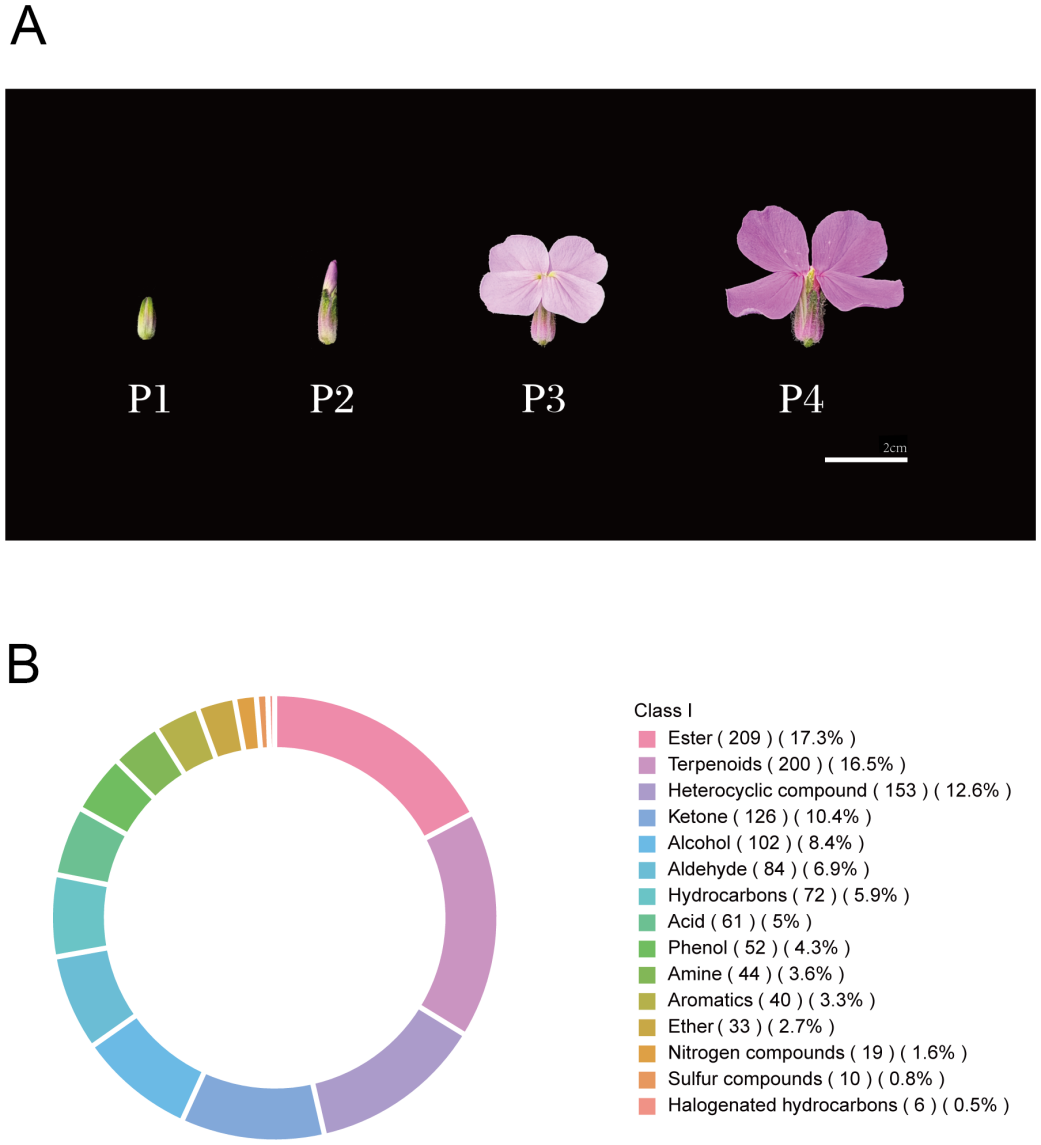
**(A)** Different flower developmental stages of *Hesperis oreophila*. P1: small bud stage; P2: large bud stage; P3: initial bloom stage; P4: full bloom stage. **(B)** Types, numbers, and proportions of volatile metabolites in *H. oreophila*.

Samples used in this study were collected from fresh flowers on the aerial parts of healthy, pest- and disease-free *H. oreophila* plants grown at the Teaching and Practice Base of Hebei Agricultural University (38°48’26.13” N, 115°24’58.30” E). This location has an annual mean temperature of 12.7 °C, annual sunshine duration of 2,560 h, annual mean precipitation of 575.4 mm, annual mean evaporation of 1,760 mm, and a frost-free period of 200 d. To investigate the biosynthetic mechanisms of floral scent compounds across flowering stages, floral organ samples of *H. oreophila* were collected at different developmental stages, including the small bud stage (P1), large bud stage (P2), initial bloom (P3), and full bloom (P4) (**Figure 1A**). All samples were collected on sunny days during the same morning time window, between 10:00 and 11:00 a.m., to minimize the effects of diurnal and environmental variation on volatile emission. During the sampling period, the environmental temperature was 25 ± 2 °C, relative humidity was 34 ± 5%, and natural light intensity was 800–1000 μmol m^−2^ s^−1^. Samples from different developmental stages were collected under the same environmental conditions to reduce systematic bias caused by short-term environmental fluctuations.

All four stages were used for HS-SPME-GC-MS-based VOC profiling. For transcriptome sequencing, only P2, P3, and P4 were selected. P1 was omitted from RNA-seq because P1 and P2 both represented bud stages and showed relatively similar VOC profiles in the metabolomic analysis, as indicated by their close clustering in PCA and hierarchical clustering. In contrast, the transition from P2 to P3 corresponded to the most pronounced shift in floral scent formation, particularly the marked increase in terpenoid VOCs. Therefore, P2 was used as the representative bud-stage transcriptomic baseline, while P3 and P4 represented the major scent-emission stages for transcriptomic comparison and integrated analysis.

Three biological replicates were collected for each developmental stage. Each biological replicate consisted of pooled floral materials from five independent, healthy, pest- and disease-free plants with similar growth status. From each plant, 3–5 flowers or floral buds at the corresponding developmental stage were collected and pooled to form one biological replicate. This sampling strategy was used to reduce the influence of individual plant variation on VOC detection and transcriptomic analysis. Immediately after collection, samples were placed into 50 mL centrifuge tubes, flash-frozen in liquid nitrogen, and stored at −80 °C until subsequent metabolomic and transcriptomic analyses. Because *H. oreophila* has been reported to emit a stronger fragrance at night, the present study characterizes VOC profiles under standardized daytime sampling conditions and may not fully represent the nocturnal emission peak.

### 2.2 RNA extraction

Total RNA was extracted using the ethanol precipitation method combined with the CTAB-PBIOZOL reagent. To ensure RNA quality, the integrity and concentration of the extracted RNA were assessed using a Qubit 4.0 fluorometer and a Qsep400 bioanalyzer.

### 2.3 HS-SPME-GC-MS analysis

Approximately 500 mg of frozen floral tissue was ground in liquid nitrogen, thoroughly homogenized, and transferred into a headspace vial. Saturated NaCl solution and 20 μL of internal standard solution were then added to each sample. The internal standard was 2-chloro-L-phenylalanine, also known as L-2-chlorophenylalanine, at a working concentration of 1 ppm (1 mg L^−1^). The samples were subjected to automated headspace solid-phase microextraction (HS-SPME) using a 120 μm DVB/CWR/PDMS SPME Arrow fiber. Samples were equilibrated at 60 °C with shaking for 5 min, followed by headspace extraction for 15 min. The fiber was subsequently desorbed at 250 °C for 5 min before GC-MS analysis. Before sample extraction, the SPME Arrow fiber was conditioned at 250 °C for 5 min in the fiber conditioning station; a new fiber was conditioned at 250 °C for 2 h before use.

VOC analysis was performed using an Agilent 8890 gas chromatograph coupled with an Agilent 5977B mass spectrometer. Separation was conducted on a DB-5MS capillary column (30 m × 0.25 mm × 0.25 μm; Agilent J&W Scientific, Folsom, CA, USA). High-purity helium (≥ 99.999%) was used as the carrier gas at a constant flow rate of 1.2 mL min^−1^. The inlet temperature was set at 250 °C, and split less injection was used with a solvent delay of 3.5 min. The oven temperature program was as follows: 40 °C for 3.5 min, increased to 100 °C at 10 °C min^−1^, increased to 180 °C at 7 °C min^−1^, and finally increased to 280 °C at 25 °C min^−1^ and held for 5 min. The ion source, quadrupole, and MS interface temperatures were set at 230 °C, 150 °C, and 280 °C, respectively. Electron impact ionization was performed at 70 eV, and selected ion monitoring (SIM) mode was used for targeted ion scanning.

VOC annotation and quantification were performed using a broad-targeted volatile metabolomics platform. Compound annotation was based on a self-built volatile metabolite database constructed from multiple plant species, published literature, partial authentic standards, and retention index information. The database contained reference retention time (RT), quantitative ions, and qualitative ions for target compounds. For each compound, one quantitative ion and two to three qualitative ions were selected. A compound was annotated when its retention time was consistent with the database reference and all selected qualitative ions were detected in the background-subtracted mass spectrum. Mass spectral matching against the NIST20 and MWGCSIM1 libraries was also used to support compound annotation, and peaks with low match quality or obvious background interference were excluded. Blank samples were used for background subtraction, and pooled quality-control (QC) samples were used to monitor instrumental stability and peak reproducibility.

Quantification was performed by integrating the selected quantitative ion of each compound, followed by correction and normalization using the internal standard and sample fresh weight. Therefore, the VOC contents reported in this study represent semi-quantitative values rather than absolute concentrations. Because authentic standards and calibration curves were not available for every detected VOC in the present analysis, the detected compounds are described as putatively annotated VOCs with high confidence rather than fully confirmed compounds. Sodium chloride was of analytical grade; n-hexane was of chromatographic grade and used for standard preparation; authentic standards used for database construction and method development were of chromatographic grade and purchased from BioBioPha or Sigma-Aldrich and were stored at −20 °C after preparation.

Orthogonal partial least squares-discriminant analysis (OPLS-DA) was performed using SIMCA-P version 14.1 (Umetrics, Umeå, Sweden) to visualize the separation of VOC profiles between developmental stages and to evaluate the relative contribution of individual variables. Before modeling, VOC abundance data were log_2_-transformed and mean-centered. For each of the six pairwise comparisons, an OPLS-DA model containing one predictive component and one orthogonal component was constructed. Model fit and predictive performance were evaluated using the *R*²X, *R*²Y, and *Q*² values generated by the software. Model robustness was further assessed using 200 permutation tests. Score plots, S-plots, and permutation plots were generated for all six pairwise comparisons.

For pairwise statistical comparisons, the resulting p-values were adjusted using the Benjamini–Hochberg false discovery rate (FDR) procedure. VOCs with a fold change of ≥2 or ≤0.5 and an FDR-adjusted *p*-value<0.05 were defined as differentially accumulated volatile organic compounds (DVOCs). Variable importance in projection (VIP) values derived from OPLS-DA were used only as supplementary indicators of the relative contribution of individual variables to group separation and were not required for DVOC identification.

### 2.4 RNA-seq and transcriptomic data analysis

RNA-seq analysis was performed for floral samples from P2, P3, and P4 only; therefore, all transcriptomic comparisons and transcriptome–metabolome integration analyses were restricted to these three stages.

Sequencing libraries were prepared using the NEBNext^®^ Ultra™ RNA Library Prep Kit for Illumina^®^ (NEB, Ipswich, MA, USA) according to the manufacturer’s instructions. cDNA was generated using the AMPure XP system (Beckman Coulter, Beverly, MA, USA) and subsequently amplified by polymerase chain reaction (PCR). Library quality was validated using an Agilent 2100 Bioanalyzer, and sequencing was performed on the Illumina NovaSeq platform at MetWare (Wuhan, China).

Raw reads were processed with fast to remove adapters and low-quality reads (sequences with > 10% N bases or > 50% bases with Q ≤ 20). In the absence of a reference genome, clean reads obtained after filtering were *de novo* assembled using Trinity to generate transcript sequences of *H. oreophila*.

To reduce transcript redundancy and obtain gene-level representative sequences, Trinity-assembled transcripts were further clustered using Corset based on read mapping information and expression patterns. Transcripts assigned to the same Corset cluster were considered to represent the same gene-level cluster, and the longest sequence within each cluster was retained as the representative unigene for downstream functional annotation and expression analysis. The completeness of the *de novo* transcriptome assembly was evaluated using BUSCO with the eukaryota_odb10 dataset, which contains 255 conserved single-copy orthologs.

Coding sequence prediction was performed using TransDecoder. First, TransDecoder.LongOrfs was used to identify candidate open reading frames, with the default minimum amino acid length set to 100 aa. To improve the reliability of CDS prediction, the predicted protein sequences were further searched against the UniProt protein database and the Pfam protein domain database. Subsequently, TransDecoder.Predict was used to integrate ORF length, coding potential, UniProt homology evidence, and Pfam domain evidence, and to retain the most reliable protein-coding regions. The predicted CDS and peptide sequences were used as supporting resources for downstream gene annotation and candidate gene characterization.

Functional annotation was performed using seven public databases: Gene Ontology (GO), Clusters of Orthologous Groups (KOG/COG), Protein Families (Pfam), Swiss-Prot (a manually annotated and reviewed protein sequence database), Kyoto Encyclopedia of Genes and Genomes (KEGG), NCBI Non-Redundant Protein Sequences (Nr) and Translated EMBL (TrEMBL, a computer-annotated protein sequence database). Gene expression abundance was estimated as FPKM values for expression visualization, PCA, clustering, and heatmap analysis. For differential expression analysis, the raw integer read-count matrix generated at the Corset-clustered unigene level, rather than FPKM values, was used as the input for DESeq2 (version 1.22.1) (Love et al., 2014). DEGs between comparison groups were identified using thresholds of false discovery rate (FDR)< 0.05 and |log_2_ fold change| > 1. GO and KEGG enrichment analyses of DEGs were performed using clusterProfiler (version 3.10.1). Expression heatmaps were generated using the Metware Cloud platform.

### 2.5 Integrated analysis of transcriptomics and volatilomics

The FPKM values of all differentially expressed genes (DEGs) in the sample groups were used for k-means clustering analysis. To further explore the associations between scent-related genes and volatile metabolites, Pearson correlation analysis was performed between the expression levels of terpenoid biosynthesis-related DEGs and the contents of selected terpenoid VOCs using R (version 4.3.2). Before correlation analysis, gene-expression and metabolite-abundance data were log_2_-transformed. Because transcriptome sequencing was conducted for P2, P3, and P4, the integrated gene–metabolite correlation analysis was restricted to these three stages. Correlations were calculated using biological replicate-level data from P2–P4 samples rather than stage mean values, resulting in nine paired observations for each gene–metabolite pair. The resulting *p*-values were adjusted using the Benjamini–Hochberg false discovery rate (FDR) method. Gene–metabolite pairs with |*r*| > 0.9 and FDR-adjusted *p* < 0.05 were retained for network construction. The gene–metabolite association network was visualized using Cytoscape (version 3.9.1). Because Pearson correlation analysis cannot establish causal regulatory relationships, the genes identified in this network were interpreted as candidate genes associated with VOC accumulation rather than confirmed regulators.

### 2.6 qRT-PCR validation of gene expression

To validate the RNA-seq expression results, 16 representative differentially expressed genes (DEGs) involved in terpenoid biosynthesis, phenylpropanoid metabolism, and fatty acid metabolism were selected for qRT-PCR analysis. These genes were selected according to the following criteria: (1) genes encoding key catalytic enzymes in core floral scent-related pathways; (2) genes showing significant differential expression patterns during flower development, including stage-specific up-regulated, down-regulated, or continuously changing genes; and (3) genes with relatively high expression abundance and significant differential expression, with FDR< 0.05 and |log_2_FC| > 1. Therefore, the selected genes represented major scent-related pathways and distinct expression patterns during flower development.

Candidate reference genes were screened based on commonly used housekeeping gene annotations and their expression stability in the RNA-seq dataset. The reference gene elongation factor 1-alpha (EF1α) was selected for relative expression normalization because it showed stable expression across P2, P3, and P4 samples, with no significant differential expression among developmental stages and low variation in FPKM values.

Total RNA used for qRT-PCR was extracted from the same batch of biological samples used for RNA-seq. Three independent biological replicates were included for each developmental stage. First-strand cDNA was synthesized using a commercial reverse transcription kit. qRT-PCR was performed using SYBR Green-based reagents on a real-time fluorescence quantitative PCR system, with four technical replicates for each biological sample. Primer sequences of the reference gene and the 16 target genes are listed in **Supplementary Table 5**. Relative gene expression levels were calculated using the 2^−ΔΔCt^ method. Statistical significance among developmental stages was evaluated using one-way ANOVA followed by Tukey’s multiple comparison test, with *p* < 0.05 considered significant. To assess concordance between qRT-PCR and RNA-seq, the mean expression value at each developmental stage (P2, P3, and P4) was calculated from the three biological replicates for each gene. The qRT-PCR relative-expression values and RNA-seq FPKM values were log_2_-transformed, and Pearson correlation coefficients (*r*) and corresponding *p*-values were calculated separately for each gene using R version 3.5.1. Because each correlation was based on three stage-level observations, the correlation coefficients were interpreted primarily as descriptive measures of agreement between the two methods.

### 2.7 Relative odor activity value analysis

Relative odor activity values (rOAVs) were calculated to estimate the potential contribution of individual VOCs to the floral aroma:


$$rOAV_{i}=\frac{C_{i}}{T_{i}}$$

Where *C_i_* is the internal-standard-normalized semi-quantitative abundance of compound *i*, and *T_i_* is its odor threshold. Threshold values were obtained from previously reported values in peer-reviewed flavor studies (Huang et al., 2022; Xue et al., 2022). Water-phase thresholds were preferentially used. When multiple values were available, the lowest reported water-phase threshold was selected to maximize screening sensitivity; compounds lacking reliable threshold data were excluded from the calculation.

Because the VOC data were semi-quantitative rather than compound-specifically calibrated absolute concentrations, rOAV was used as a relative screening index. Compounds with rOAV ≥ 1 were considered potential aroma contributors rather than confirmed aroma-contributing compounds.

## 3 Results

### 3.1 Composition and content of floral volatile compounds in *Hesperis oreophila*

GC-MS analysis of volatile metabolites across four flower developmental stages (P1, P2, P3, and P4) of *H. oreophila* detected a total of 1,211 volatile organic compounds (VOCs). Based on their chemical properties, these compounds were classified into 15 major categories, including 200 terpenoids (16.52%), 209 esters (17.26%), 126 ketones (10.4%), 102 alcohols (8.42%), 52 phenols (4.29%), 33 ethers (2.73%), 84 aldehydes (6.94%), 61 acids (5.04%), 44 amines (3.63%), 72 hydrocarbons (5.95%), 6 halogenated hydrocarbons (0.5%), 40 aromatic hydrocarbons (3.3%), 153 heterocyclic compounds (12.63%), 19 nitrogen-containing compounds (1.57%), and 10 sulfur-containing compounds (0.83%). Among them, esters, terpenoids, heterocyclic compounds, and ketones accounted for 17.26%, 16.52%, 12.63%, and 10.4%, respectively, together representing 56.81% of the total metabolites. In terms of both compound number and abundance, these four categories constituted the major metabolites (**Figure 1B**).

The total content of volatile metabolites (VOCs) in flowers exhibited an initial decrease followed by an increase, reaching a maximum of 464.74 μg·g^−1^ at the full bloom stage. At the small bud and large bud stages, ketones were the most abundant VOCs, accounting for 23.86% and 25.05%, respectively, followed by esters at 20.72% and 21.58%, respectively. At the initial bloom and full bloom stages, terpenoids were the most abundant VOCs, accounting for 31.80% and 35.77%, respectively, followed by esters (13.33% and 12.16%), aldehydes (11.63% and 11.04%), and ketones (10.96% and 9.59%). These four compound classes were therefore considered the major constituents of the floral scent of *H. oreophila*. Analysis of volatile compound contents across different flower developmental stages (**Table 1**) revealed a notable finding: terpenoid content changed markedly from the initial bloom stage onward and peaked at 166.25 μg·g^−1^ at full bloom, a trend consistent with the variation in total VOC content. This change in terpenoid abundance is presumed to be the primary factor responsible for the shift in floral scent profile.

**Table 1:** Contents of various volatile compounds in *Hesperis oreophila* at different flowering stages.

Compound class	Content at P1 (μg·g^−1^)	Content at P2 (μg·g^−1^)	Content at P3 (μg·g^−1^)	Content at P4 (μg·g^−1^)
Esters	27.37 ± 3.69 **a**	23.07 ± 3.11 **a**	39.00 ± 5.41 **ab**	56.49 ± 16.02 **b**
Heterocyclic compounds	18.97 ± 0.33 **a**	13.89 ± 2.10 **a**	17.77 ± 2.30 **a**	23.77 ± 7.09 **a**
Ketones	31.51 ± 1.03 **a**	26.78 ± 4.10 **a**	32.06 ± 3.69 **a**	44.57 ± 14.76 **a**
Hydrocarbons	3.79 ± 0.23 **ab**	3.21 ± 0.46 **a**	4.60 ± 0.60 **ab**	5.97 ± 1.78 **b**
Terpenoids	6.75 ± 0.88 **a**	5.71 ± 0.48 **a**	93.02 ± 11.60 **b**	166.25 ± 41.56 **c**
Acids	4.98 ± 0.76 **ab**	4.38 ± 0.59 **a**	5.80 ± 0.77 **ab**	8.61 ± 2.65 **b**
Aldehydes	16.07 ± 0.18 **ab**	12.64 ± 2.19 **a**	34.01 ± 4.52 **bc**	51.32 ± 13.82 **c**
Ethers	0.23 ± 0.02 **a**	0.21 ± 0.03 **a**	2.13 ± 0.36 **b**	3.58 ± 1.08 **b**
Halogenated hydrocarbons	0.07 ± 0.01 **a**	0.08 ± 0.01 **a**	1.46 ± 0.16 **b**	0.83 ± 0.63 **ab**
Sulfur-containing compounds	0.57 ± 0.03 **a**	0.36 ± 0.07 **a**	0.65 ± 0.15 **ab**	1.21 ± 0.39 **b**
Nitrogen-containing compounds	0.25 ± 0.02 **a**	0.38 ± 0.05 **a**	15.85 ± 1.85 **b**	26.96 ± 6.61 **c**
Phenols	1.06 ± 0.13 **a**	1.07 ± 0.11 **a**	13.39 ± 2.18 **b**	24.61 ± 6.68 **c**
Aromatic hydrocarbons	0.24 ± 0.00 **a**	0.27 ± 0.06 **a**	1.14 ± 0.16 **a**	2.21 ± 0.69 **b**
Alcohols	17.00 ± 0.47 **a**	12.20 ± 1.38 **a**	22.86 ± 2.77 **ab**	34.61 ± 9.94 **b**
Amines	3.21 ± 0.05 **a**	2.64 ± 0.37 **a**	8.82 ± 1.20 **b**	13.75 ± 3.59 **b**
Total	132.07 ± 7.62 **ab**	106.90 ± 15.70 **a**	292.55 ± 38.26 **bc**	464.74 ± 124.87 **c**

Data are semi-quantitative values presented as mean ± SD. Different lowercase letters in the same row indicate significant differences among different flowering stages at *P* < 0.05 (Tukey’s HSD test).

All 1,211 metabolites were detected at each developmental stage, albeit at varying levels. To cover as many floral scent components as possible, volatile compounds with relatively high relative contents were selected from each stage. Based on this principle, a total of 31 volatile compounds were screened from stage P1, 22 from stage P2, 57 from stage P3, and 73 from stage P4.

**Figure 2:**
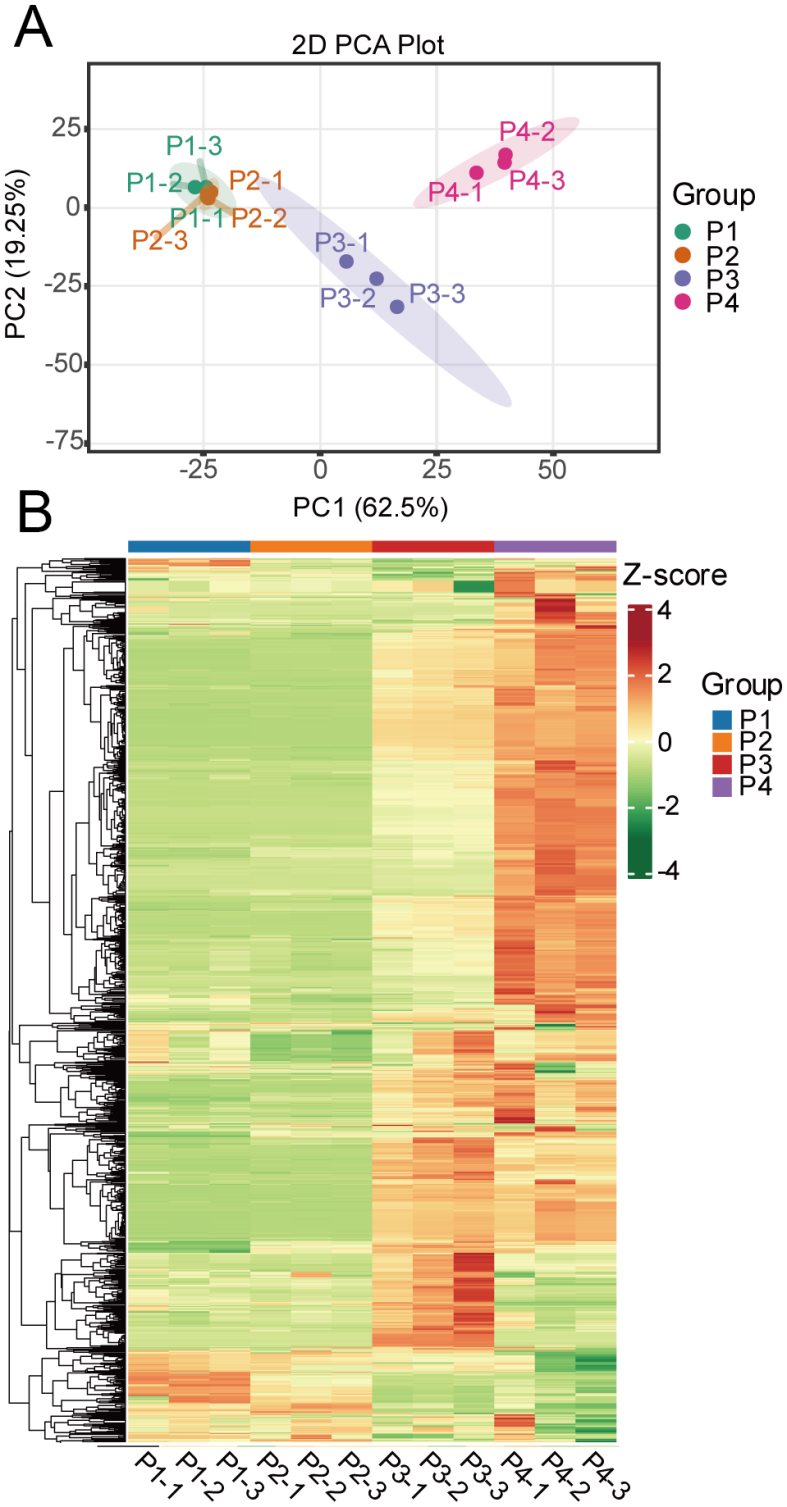
**(A)** Principal component analysis (PCA) plot of volatile metabolites in *Hesperis oreophila* at different flower developmental stages. **(B)** Hierarchical clustering heatmap of metabolites in *H. oreophila* at different flower developmental stages.

PCA analysis (**Figure 2A**) showed that the first principal component (PC1) explained 62.5% of the total variance, and the second principal component (PC2) explained 19.25%. The P3 group was positioned between the P2 and P4 groups, consistent with the progression of flower development. The three replicate samples from each stage clustered together. The P1 and P2 groups were relatively close to each other along PC1 and could be clearly separated from the P3 and P4 samples, indicating that the compositional differences in volatile metabolites between P1 and P2 samples were relatively small, and these differences gradually increased as flowering progressed. Hierarchical cluster analysis of all samples showed similar results (**Figure 2B**): P1 and P2 samples clustered together first and then joined the P3 and P4 samples. Collectively, these results demonstrate that the differences in VOCs among floral organs of *H. oreophila* gradually increased with the progression of flower development. Notably, P1 and P2 were both bud-stage samples and clustered closely in the metabolomic PCA and hierarchical clustering analyses, indicating that their VOC profiles were relatively similar. By contrast, the transition from P2 to P3 showed a marked divergence in VOC accumulation, consistent with the onset of active floral scent formation. This pattern provided a metabolomic basis for selecting P2, P3, and P4 for transcriptome sequencing and integrated analysis.

### 3.2 rOAV profiling of floral volatiles in *Hesperis oreophila*

**Figure 3:**
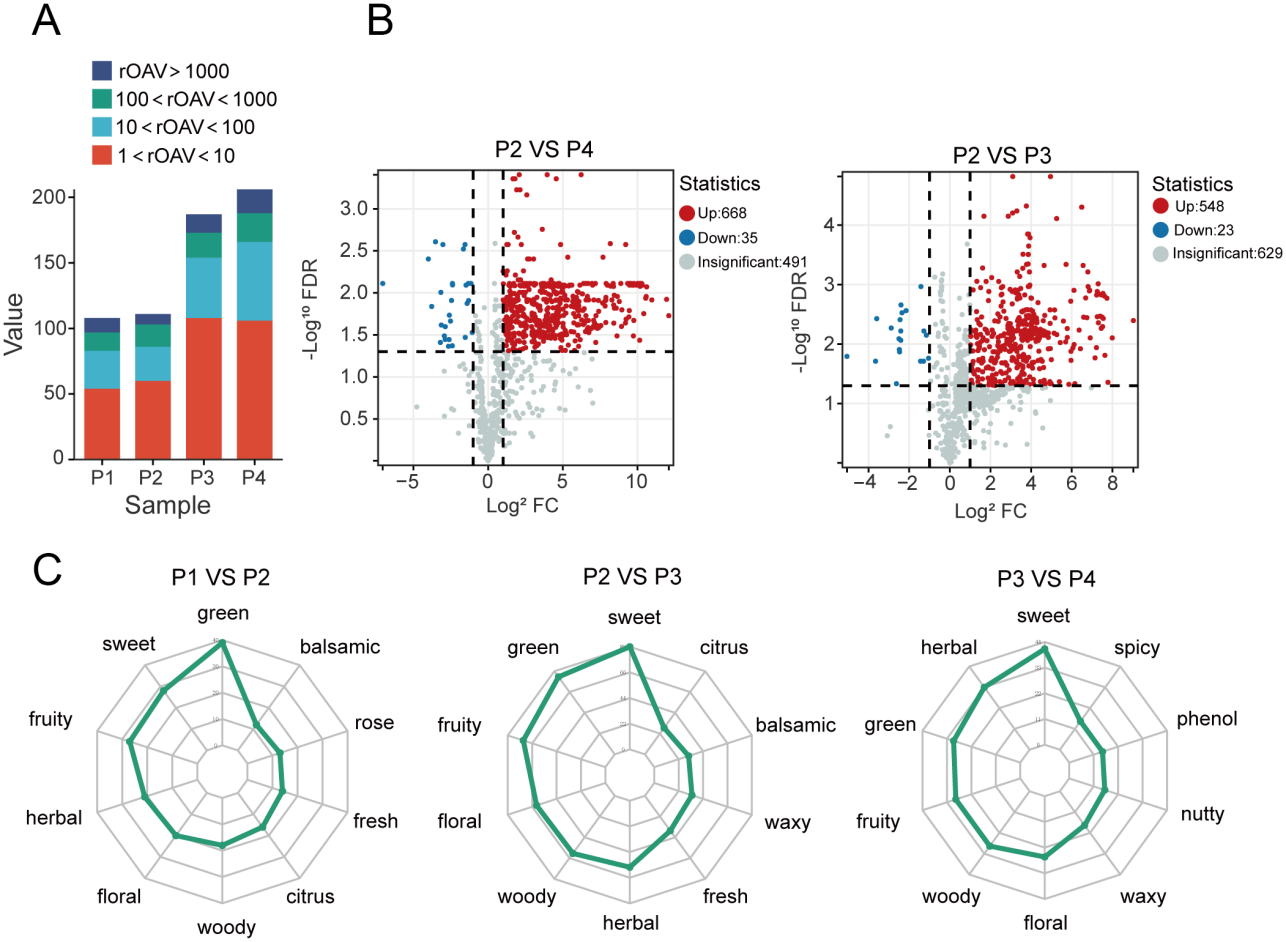
**(A)** Stacked bar chart of the numbers of putative aroma-contributing VOCs at each rOAV level across different flowering stages. **(B)** Volcano plot of differential metabolites. **(C)** Radar chart showing thedistribution of DVOCs among literature-reported odor-description categories.

Quantitative VOC profiling alone cannot fully determine the sensory contribution of individual volatile compounds. Therefore, rOAV analysis was used as a literature-threshold-based approach to preliminarily estimate the potential aroma contribution of detected VOCs. We calculated the rOAV of VOCs at different flower developmental stages (**Figure 3A**). In this study, VOCs with rOAV ≥ 1 were considered putative aroma-contributing compounds, whereas VOCs with rOAV > 10 were considered to have relatively high potential contribution to the overall aroma profile. The number of VOCs with rOAV ≥ 1 showed a continuous increasing trend from the small bud stage to the full bloom stage, with 108, 111, 187, and 206 compounds identified across the four stages, respectively. These VOCs were classified into 14 categories. At the small bud and large bud stages, aldehydes were the most abundant category, with 24 and 26 compounds, respectively. At the initial bloom and full bloom stages, terpenoids were the most abundant category, with 32 and 37 compounds, respectively.

### 3.3 Analysis of differential metabolites at different flowering stages

To characterize developmental differences in VOC profiles, OPLS-DA was performed for all six pairwise comparisons among the four flowering stages: P1 vs. P2, P1 vs. P3, P1 vs. P4, P2 vs. P3, P2 vs. P4, and P3 vs. P4. Each model contained one predictive component and one orthogonal component. The OPLS-DA score plots showed clear separation between the compared developmental stages, indicating pronounced differences in their VOC profiles. All models showed *R*²X and *R*²Y values greater than 0.8 and *Q*² values greater than 0.9, indicating good model fit and predictive performance. The results of the 200 permutation tests further supported the robustness of the observed group separation. The corresponding score plots, S-plots, and permutation plots are provided in **Supplementary Figure 1**–**3**.

To avoid identifying DVOCs solely on the basis of supervised multivariate modeling, differential VOC screening was based on fold change and FDR-adjusted statistical significance. VOCs with a fold change of ≥2 or ≤0.5 and an FDR-adjusted *p*-value<0.05 were classified as DVOCs. VIP values were retained only as supplementary indicators of variable contribution and were not used as mandatory screening criteria.

The six pairwise comparisons yielded different numbers of DVOCs. Relative to P1, 196, 621, and 703 DVOCs were identified in P1 vs. P2, P1 vs. P3, and P1 vs. P4, respectively, indicating that differences in VOC profiles generally increased as flowering progressed. Among all comparisons, P2 vs. P4 yielded the largest number of DVOCs, with 708 compounds, including 682 with increased abundance and 26 with decreased abundance. Among adjacent developmental stages, the greatest difference was observed between P2 and P3, with 571 DVOCs, of which 548 increased and 23 decreased in abundance (**Figure 3B**). The remaining pairwise comparisons are summarized in **Supplementary Table 7**. Notably, VOCs with increased abundance outnumbered those with decreased abundance in every comparison, indicating widespread VOC accumulation during flower opening that may collectively contribute to the differentiation of stage-specific floral scent profiles.

Moreover, analysis of volatile organic compounds by aroma type (**Figure 3C**), based on the sensory classification framework of Chen et al. (2025), revealed that, from P1 to P2, compounds associated with green descriptors showed the largest relative changes. From P2 to P3, the representation of compounds associated with green and sweet descriptors increased, with sweet-associated compounds accounting for a prominent proportion of the DVOCs. From P3 to P4, compounds associated with sweet descriptors remained relatively abundant, while compounds associated with herbal, woody, and other descriptors became more diverse. These categories represent literature-based descriptive groupings and should not be interpreted as direct sensory measurements of *H. oreophila.*

Analysis of the top 10 metabolites ranked by fold change in each comparison group (**Supplementary Table 1**) revealed a total of 40 compounds, which were classified into nine categories. These categories were terpenoids, heterocyclic compounds, amines, phenols, esters, aromatic hydrocarbons, ethers, ketones, and nitrogen-containing compounds. Among them, terpenoids were the most abundant, with 15 compounds, followed by heterocyclic compounds with six compounds; nitrogen-containing compounds were the least abundant, with only one compound. In the P1 vs. P2 comparison, seven of the top 10 metabolites were classified as terpenoids: Bicyclo[3.1.0]hex-2-ene, 4-methylene-1-(1-methylethyl)-; Camphene; α-Pinene; (1R)-2,6,6-Trimethylbicyclo[3.1.1]hept-2-ene; (1S)-2,6,6-Trimethylbicyclo[3.1.1]hept-2-ene; Bicyclo[3.1.0]hex-2-ene, 2-methyl-5-(1-methylethyl)-; and Myroxide.

### 3.4 RNA-seq analysis of floral organs at P2–P4 stages

To explore the DEGs associated with the major scent-transition stages of *H. oreophila*, nine cDNA libraries were constructed using total RNA extracted from flowers at the large bud stage (P2), initial bloom stage (P3), and full bloom stage (P4) on the Illumina NovaSeq platform, with three biological replicates per stage. A total of 61.16 Gb of clean data was obtained, with each sample yielding at least 5 Gb of clean data and a Q30 base percentage of 88% or higher. From the nine samples, a total of 456,494,684 raw reads and 407,709,454 clean reads were obtained from the nine libraries. The mean percentages of Q20 and Q30 bases were 95.81% and 88.44%, respectively (**Supplementary Table 2**). These findings confirmed the high quality of the transcriptomic data, making them suitable for downstream analyses.

Trinity initially generated 171,601 transcripts, which were subsequently clustered using Corset to obtain 107,689 representative unigenes. The total assembly size was 153,235,649 bp, with an N50 of 1,926 bp and an N90 of 704 bp. The mean unigene length was 1,423 bp.

To further assess assembly completeness beyond length-based statistics, BUSCO analysis was performed using the eukaryota_odb10 dataset containing 255 conserved orthologs. The assembly contained 252 complete BUSCOs, corresponding to 98.82% completeness, including 25 single-copy BUSCOs (9.80%) and 227 duplicated BUSCOs (89.02%). Only two BUSCOs were fragmented (0.78%), and one BUSCO was missing (0.39%). The high proportion of complete BUSCOs indicated that the transcriptome assembly captured most conserved gene space. The relatively high duplicated BUSCO proportion may reflect transcript redundancy, alternative isoforms, and closely related paralogous transcripts, which are commonly observed in *de novo* transcriptome assemblies. Therefore, Corset clustering was used to reduce redundancy and to define representative unigenes for downstream analysis.

Trinity assembly generated 171,601 transcript sequences, with a mean length of 1,211 bp, an N50 of 1,779 bp, an N90 of 577 bp, and a total length of 207,886,307 bp. After Corset hierarchical clustering and representative sequence selection, 107,689 unigenes were retained, with a mean length of 1,423 bp, an N50 of 1,926 bp, an N90 of 704 bp, and a total length of 153,235,649 bp. These results indicate that the *de novo* transcriptome assembly had high completeness and acceptable continuity, providing a suitable basis for downstream functional annotation and candidate gene mining.

### 3.5 Gene annotation and functional classification

CDS prediction was further performed using TransDecoder to support protein-coding gene characterization. Candidate ORFs were first identified using TransDecoder.LongOrfs, and the predicted protein sequences were compared against the UniProt and Pfam databases to incorporate homology and conserved domain evidence. TransDecoder.Predict was then used to retain the most reliable coding regions. The predicted CDS and peptide sequence files were generated for downstream annotation and candidate gene analysis. CDS and peptide files were provided in the NCBI Sequence Read Archive under BioProject accession number: PRJNA1493792, with SRA accession number: SRR39600458–SRR39600466.

Functional annotation showed that 90,228 of 107,689 unigenes were annotated in at least one public database (**Supplementary Table 3**), accounting for 83.79% of the total unigene set. Specifically, 67,594 unigenes (62.77%) were annotated in KEGG, 88,378 (82.07%) in NR, 68,858 (63.94%) in Swiss-Prot, 88,235 (81.94%) in TrEMBL, 56,537 (52.50%) in KOG, 76,904 (71.41%) in GO, and 61,025 (56.67%) in Pfam. The high overall annotation rate supported the reliability of downstream pathway enrichment and candidate gene screening. In particular, the KEGG annotation coverage provided a useful basis for identifying genes involved in terpenoid, phenylpropanoid/benzenoid, and fatty-acid-derived volatile biosynthesis.

**Figure 4:**
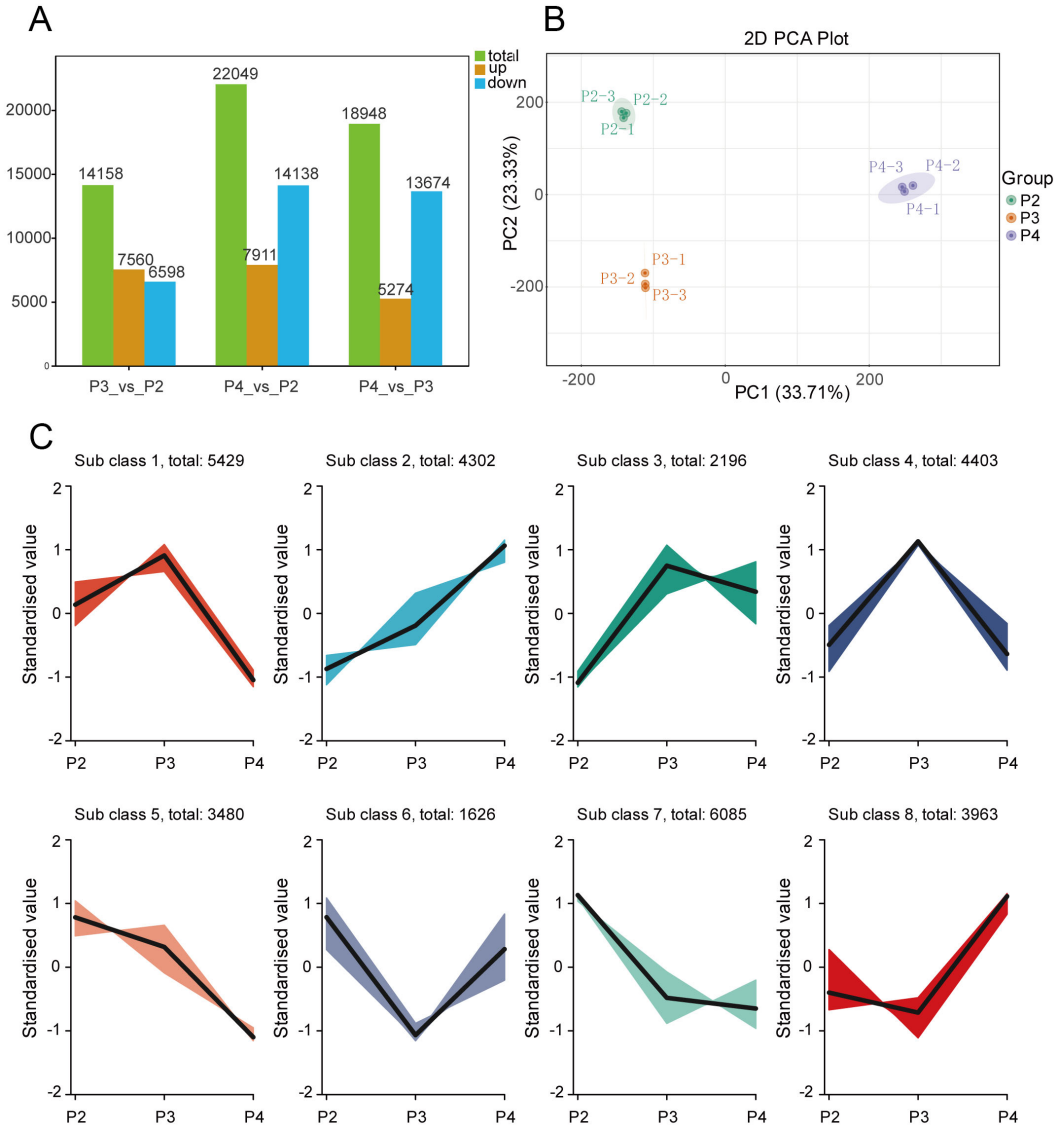
**(A)** Bar chart showing the number of DEGs in each comparison group. **(B)** PCA plot of different samples. **(C)** K-means clustering plot of DEGs.

FPKM values were used for expression visualization, whereas differential expression analysis was performed with DESeq2 using the raw integer count matrix with the following significance thresholds: false discovery rate (FDR, adjusted *p*-value)< 0.05 and |log_2_ fold change| ≥ 1. A total of 31,485 DEGs were identified across all comparisons. The numbers of DEGs in each comparison group are shown in the figure. The overall expression trends among samples were largely consistent with those of the VOCs. Compared with P2, 14,158 genes were differentially expressed at P3, of which 7,560 were up-regulated and 6,598 were down-regulated. Compared with P3, 18,948 genes were differentially expressed at P4, of which 5,274 were up-regulated and 13,674 were down-regulated. As flowering progressed, the number of DEGs between groups gradually increased, and the largest number of DEGs was observed between P2 and P4 (**Figure 4A**). This pattern indicates that significant differences in gene expression occurred across flowering stages, showing an upward trend consistent with the observed variation in VOCs.

PCA based on FPKM values showed similar results (**Figure 4B**): the biological replicates of P4 samples clustered together and were clearly separated from the other two sample groups. These results indicate that the differences in gene expression in *H. oreophila* increased during flowering.

To investigate the expression patterns of differentially expressed genes in *H. oreophila*, k-means clustering based on FPKM values was performed on all DEGs. The DEGs were classified into eight subclusters across the flowering stages, indicating that genes within each cluster showed similar developmental expression patterns.

The total number of genes in each subcluster differed markedly (**Figure 4C**). Subcluster 7, containing 6,085 genes, was the largest subcluster and exhibited an overall downward trend. Subcluster 6, containing only 1,626 genes, was the smallest subcluster and showed a dynamic pattern of initial decline followed by increase. Subclusters 1, 3, and 4 contained 5,429, 2,196, and 4,403 genes, respectively, and all displayed a dynamic pattern of initial increase followed by decline, although the magnitude of change varied among them. Subcluster 5, with 3,480 genes, exhibited a continuous downward trend. In contrast, Subcluster 2 showed the opposite dynamic pattern, with its 4,302 genes continuously increasing. Subcluster 8, containing 3,963 genes, displayed a pattern of initial decline followed by increase. These results reflect the heterogeneity in expression regulation patterns among the DEGs.

### 3.6 Differentially expressed genes in scent-related pathways

**Figure 5:**
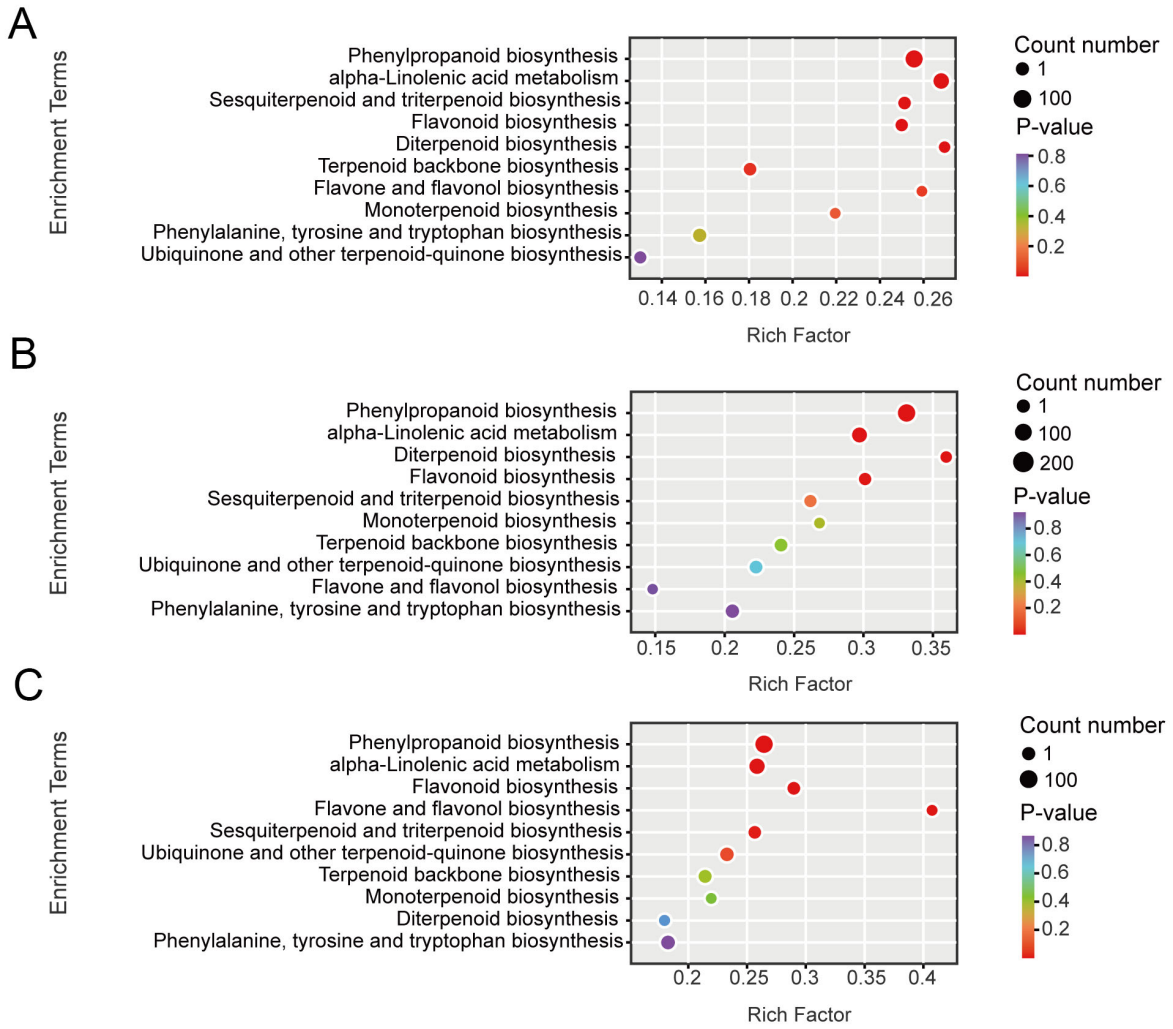
KEGG enrichment scatter plot of differentially expressed genes (DEGs) in comparison groups at different flower developmental stages of *Hesperis oreophila*. P2 vs P3 **(A)**; P3 vs P4 **(B)**; P2 vs P4 **(C)**. Pathways with significant enrichment are highlighted in purple, and the same pathways are marked with the same color for comparison purposes. The rich factor is defined as the ratio of the number of DEGs associated with a given pathway to the total number of annotated unigenes assigned to that pathway in the background unigene set.

Plant floral scent compounds include terpenoids, phenylpropanoids/benzenoids, and fatty acids and their derivatives. KEGG pathway enrichment analysis was performed for each comparison (P2 vs. P3, P3 vs. P4, and P2 vs. P4) to identify the scent-related metabolic pathways involving DEGs. KEGG analysis was conducted on 11 pathways potentially involved in floral scent formation, including “Terpenoid backbone biosynthesis” (ko00900), “Monoterpenoid biosynthesis” (ko00902), “Diterpenoid biosynthesis” (ko00904), “Sesquiterpenoid and triterpenoid biosynthesis” (ko00909), “Phenylpropanoid biosynthesis” (ko00940), “Flavonoid biosynthesis” (ko00941), “Flavone and flavonol biosynthesis” (ko00944), “Phenylalanine, tyrosine, and tryptophan biosynthesis” (ko00400), “alpha-Linolenic acid metabolism” (ko00592), “Fatty acid biosynthesis” (ko00061), and “Ubiquinone and other terpenoid-quinone biosynthesis” (ko00130). The KEGG enrichment results of DEGs among the comparison groups revealed that the phenylpropanoid biosynthesis pathway, alpha-linolenic acid metabolism pathway, and flavonoid biosynthesis pathway were consistently and significantly enriched in all comparisons (*p*-value< 0.05). In addition, in the comparison between P2 and P3 (**Figure 5A**), DEGs associated with diterpenoid biosynthesis and sesquiterpenoid and triterpenoid biosynthesis pathways were significantly enriched, with enrichment factors of 0.27 and 0.25 and *p*-values of 0.0052 and 0.00010, respectively. However, in the comparison between P3 and P4 (**Figure 5B**), the enrichment factor and significance of the diterpenoid biosynthesis pathway both declined, whereas sesquiterpenoid and triterpenoid biosynthesis remained significantly enriched (*p*-value< 0.05). In contrast, the flavone and flavonol biosynthesis pathway was not significantly enriched in the P2 vs. P3 comparison (enrichment factor: 0.26, *p*-value: 0.09) but showed markedly increased enrichment in the P3 vs. P4 comparison (enrichment factor: 0.41, *p*-value: 0.01) (**Figure 5B**). These results suggest that the genes involved in “Phenylpropanoid biosynthesis” (ko00940), “alpha-Linolenic acid metabolism” (ko00592), and “Sesquiterpenoid and triterpenoid biosynthesis” (ko00909) may play important roles in aroma-related biosynthesis, particularly in terpenoid and phenylpropanoid metabolism. Although the terpenoid backbone biosynthesis and monoterpenoid biosynthesis pathways were not significantly enriched in all pairwise transcriptomic comparisons, this result should be interpreted with caution and does not weaken the metabolic evidence for the predominant contribution of terpenoids to floral scent formation in *H. oreophila*. In the present study, the metabolomic dataset provided direct chemical evidence that terpenoids became the major VOC class after flower opening, accounting for 31.80% and 35.77% of total VOC abundance at P3 and P4, respectively, and reaching the highest content of 166.25 μg g^−1^ at full bloom. By contrast, KEGG enrichment analysis evaluates whether DEGs are statistically overrepresented in a given pathway relative to the background gene set, but it does not directly quantify pathway activity, metabolic flux, product accumulation, or volatile emission. Therefore, the absence of significant enrichment at the whole-pathway level does not necessarily indicate low biological relevance of terpenoid metabolism. For volatile terpenoid biosynthesis, changes in a limited number of rate-limiting enzymes, branch-point enzymes, or terpene synthases, such as *DXS*, *MVK*, *GPPS*/*GGPPS*, and specific *TPS* members, may be sufficient to redirect precursor flow and promote downstream terpenoid accumulation. Moreover, terpenoid abundance is also shaped by precursor availability, enzyme catalytic efficiency, substrate specificity, subcellular compartmentalization between the MVA and MEP pathways, post-transcriptional regulation, and emission dynamics. Thus, the high proportional abundance of terpenoid VOCs at P3/P4, together with the stage-specific expression of terpenoid-related genes, supports the interpretation that terpenoid accumulation at P3 and P4 may be associated with expression changes in a limited number of precursor-related or product-specific biosynthetic genes rather than with significant enrichment of the entire pathway.

**Figure 6:**
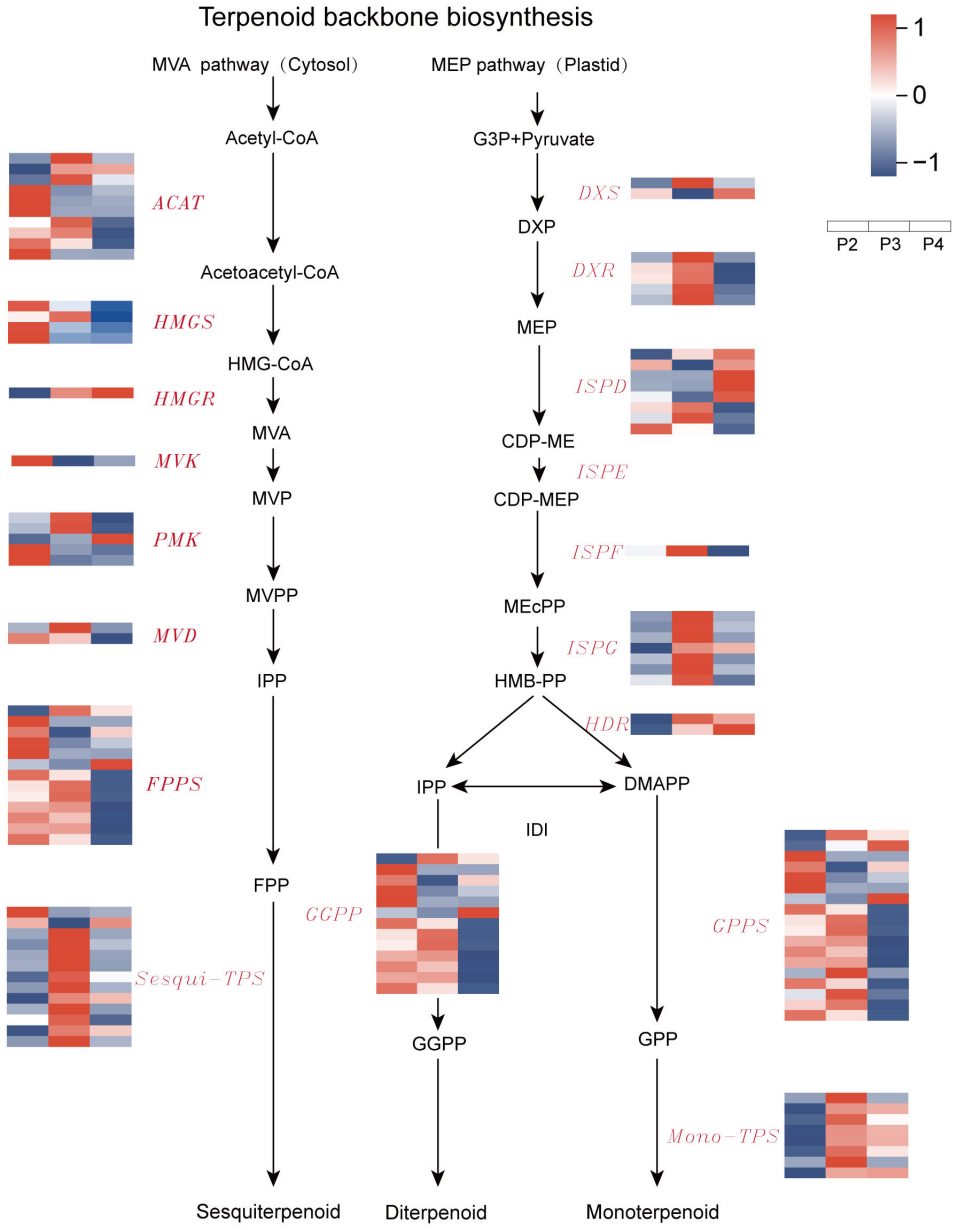
Overview of terpenoid precursor and terpene biosynthetic pathways. Black bold font represented abbreviations of compounds. Red bold italicized font represented abbreviations of coding genes: *ACAT*, acetyl-CoA acetyltransferase; *HMGS*, 3-hydroxy-3-methylglutaryl-CoA synthase; *HMGR*, 3-hydroxy-3-methylglutaryl-CoA reductase; *MVK*, mevalonate kinase; *PMK*, phosphomevalonate kinase; *MVD*, mevalonate diphosphate decarboxylase; *FPPS*, farnesyl diphosphate synthase; *DXS*, 1-deoxy-D-xylulose-5-phosphate synthase; *DXR*, 1-deoxy-D-xylulose-5-phosphate reductoisomerase; *ISPD*, 2-C-methyl-D-erythritol 4-phosphate cytidylyltransferase; *ISPE*, 4-diphosphocytidyl-2-C-methyl-D-erythritol kinase; *ISPF*, 2-C-methyl-D-erythritol 2,4-cyclodiphosphate synthase; *ISPG*, (E)-4-hydroxy-3-methylbut-2-enyl-diphosphate synthase; *HDR*, 4-hydroxy-3-methylbut-2-enyl diphosphate reductase; *GPPS*, geranyl diphosphate synthase; *GGPPS*, geranylgeranyl diphosphate synthase; *Mono-TPS*, monoterpene synthase; *Sesqui-TPS*, sesquiterpene synthase.

The major volatile components of *H. oreophila* belong to terpenoids and phenylpropanoids; therefore, we analyzed the gene expression patterns of terpenoid and phenylpropanoid pathways (**Figure 6**). Terpenoids represent the largest category of floral scent volatile metabolites. The terpenoid backbone biosynthesis pathway comprises the MVA and MEP pathways. Based on the transcriptomic data, 36 and 56 DEGs were annotated in these two pathways, respectively, including 10 acetyl-CoA acetyltransferase (*ACAT*) genes, 4 3-hydroxy-3-methylglutaryl-CoA synthase (*HMGS*) genes, 1 3-hydroxy-3-methylglutaryl-CoA reductase (*HMGR*) gene, 1 mevalonate kinase (*MVK*) gene, 5 phosphomevalonate kinase (*PMK*) genes, 2 mevalonate diphosphate decarboxylase (*MVD*) genes, 13 farnesyl diphosphate synthase (*FPPS*) genes, 2 1-deoxy-D-xylulose-5-phosphate synthase (*DXS*) genes, 5 1-deoxy-D-xylulose-5-phosphate reductoisomerase (*DXR*) genes, 8 2-C-methyl-D-erythritol 4-phosphate cytidylyltransferase (*ISPD*) genes, 1 2-C-methyl-D-erythritol 2,4-cyclodiphosphate synthase (*ISPF*) gene, 7 (E)-4-hydroxy-3-methylbut-2-enyl-diphosphate synthase (*ISPG*) genes, 2 4-hydroxy-3-methylbut-2-enyl diphosphate reductase (*HDR*) genes, 18 geranyl diphosphate synthase (*GPPS*) genes, 13 geranylgeranyl diphosphate synthase (*GGPPS*) genes, and 21 terpene synthase (*TPS*) genes. Notably, the majority of DEGs in these two pathways exhibited opposite expression trends. In the MVA pathway, 20 genes, including *ACAT*, *HMGS*, *HMGR*, *PMK*, *MVD*, *FPPS*, and *TPS*, showed high transcript levels at the large bud stage and then gradually declined. In the MEP pathway, 37 genes, including *DXS*, *DXR*, *ISPD*, *ISPF*, *ISPG*, *HDR*, *GPPS*, *GGPPS*, and *TPS*, showed high transcript levels at the initial bloom stage. Furthermore, among the *TPS* genes identified in these two pathways, only one exhibited high transcript levels at the large bud stage, whereas all the others showed high transcript levels at the initial bloom stage.

In addition, the phenylpropanoid biosynthesis pathway was annotated with 142 DEGs encoding six known enzymes, and these DEGs exhibited distinct expression patterns across different flower developmental stages: 22 DEGs were highly expressed at the large bud stage, 73 DEGs at the initial bloom stage, and 43 DEGs at the full bloom stage (**Figure 6**).

The majority of the DEGs enriched in the two scent-related pathways described above exhibited high transcript levels at the initial bloom stage. Combined with the observation that volatile metabolites increased substantially at this stage, it is inferred that the initial bloom stage represents a major developmental transition for floral scent formation.

### 3.7 Validation of representative gene expression by qRT-PCR

**Figure 7:**
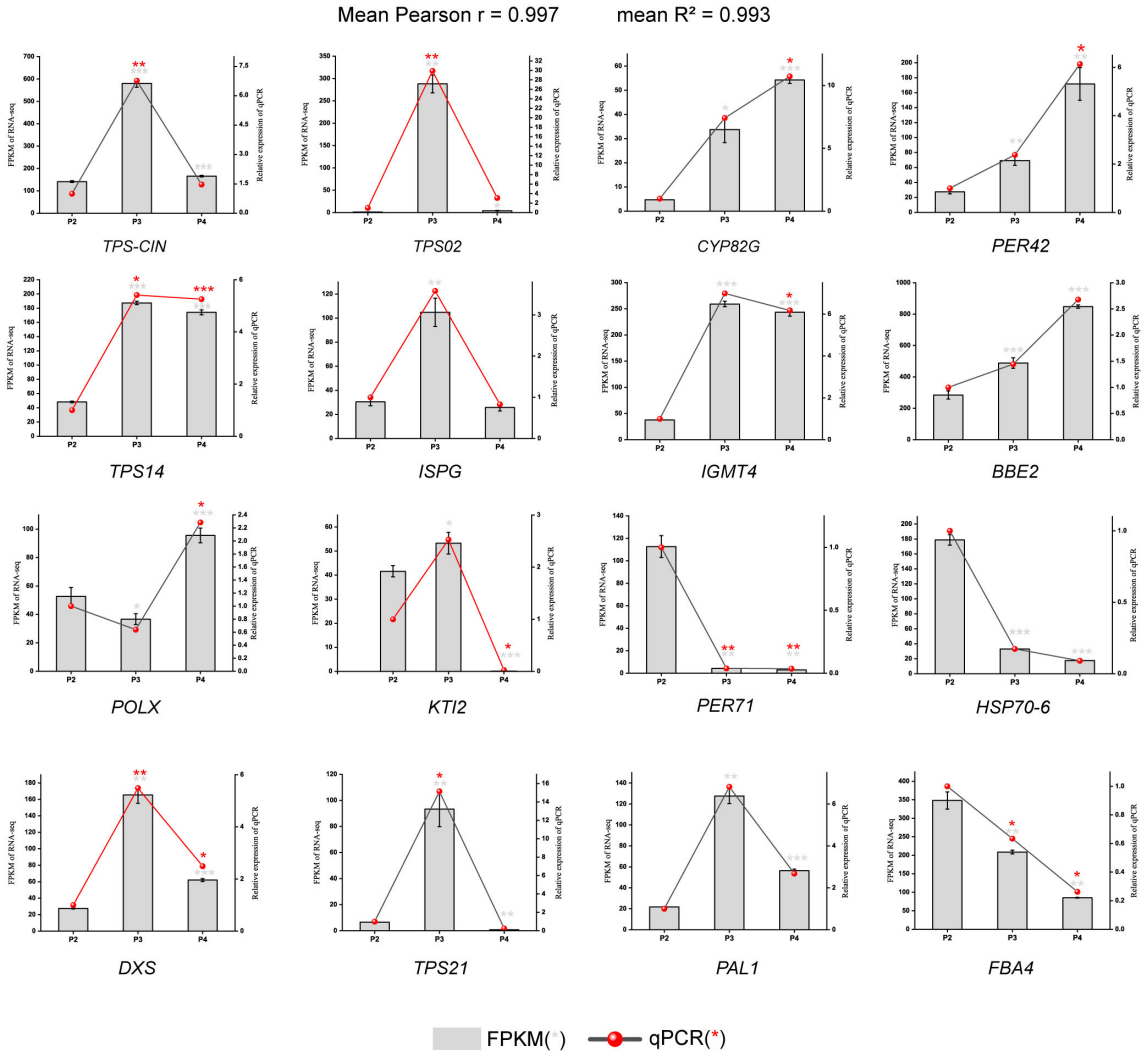
Validation by quantitative real-time PCR (qRT-PCR). Red lines and points indicate RNA-seq FPKM values plotted against the left y-axis; gray bars indicate qRT-PCR relative expression plotted against the right y-axis. Values are means ± SD of three biological replicates. Statistical differences among stages for qRT-PCR data were evaluated by one-way ANOVA followed by Tukey’s HSD test. One asterisk: *p*-value was less than 0.05; Two asterisks: *p*-value was less than 0.01. *TPS-CIN*, terpene synthase CIN; *TPS02*, terpene synthase 02; *CYP82G*, cytochrome P450 82G; *PER42*, peroxidase 42; *TPS14*, terpene synthase 14; *ISPG*, (E)-4-hydroxy-3-methylbut-2-enyl-diphosphate synthase; *IGMT4*, indole glucosinolate O-methyltransferase 4; *BBE2*, berberine bridge enzyme 2; *POLX*, polymerase X; *KTI2*, trypsin inhibitor 2; *PER71*, peroxidase 71; *HSP70-6*, heat shock protein 70-6; *DXS*, 1-deoxy-D-xylulose-5-phosphate synthase; *TPS21*, terpene synthase 21; *PAL1*, phenylalanine ammonia-lyase 1; *FBA4*, fructose-bisphosphate aldolase 4. Gene-specific Pearson correlation coefficients and corresponding *p*-values for the agreement between qRT-PCR and RNA-seq expression patterns are provided in **Supplementary Table 6**.

To validate the transcriptomic data, we selected 16 representative DEGs involved in major floral-scent-related pathways and displaying distinct developmental expression patterns, and analyzed their expression in P2, P3, and P4 by qRT-PCR. The expression patterns determined by qRT-PCR were generally consistent with those obtained by RNA-seq (**Figure 7**). Pearson correlation analysis based on the log_2_-transformed stage means further showed that the gene-specific correlation coefficients ranged from 0.988 to 1.000, with a mean *r* of 0.997 and a mean coefficient of determination (*R*²) of 0.993. The corresponding *r* and *p*-values for all 16 genes are provided in **Supplementary Table 6**. Given that each correlation was based on only three developmental stages, these results were interpreted primarily as descriptive evidence of agreement between the two expression methods.

### 3.8 Integrated analysis of transcriptomics and metabolomics

Considering the dominant role of terpenoids in *H. oreophila*, Pearson correlation analysis was employed to explore associations between terpenoid volatile metabolites and key structural genes. This analysis focused on DEGs related to terpenoid biosynthesis and 20 volatile metabolites with rOAV > 1 at the initial bloom and full bloom stages, using thresholds of |*r*| > 0.9 and FDR-adjusted *p* < 0.05 to identify highly correlated gene–metabolite pairs. Correlations were calculated using biological replicate-level data from P2–P4 samples.

**Figure 8:**
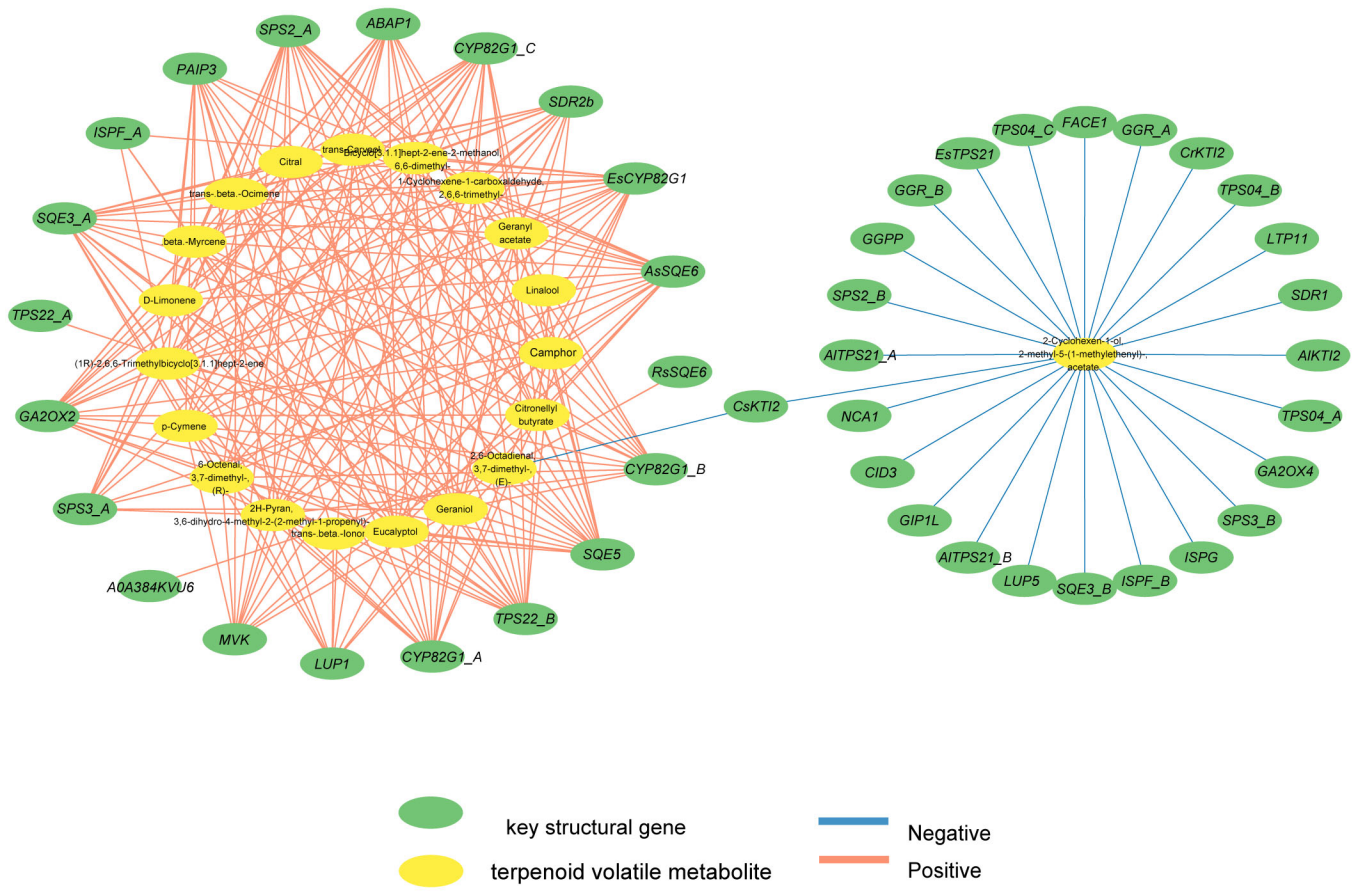
Gene–metabolite association network diagram. Green circles represent key structural genes; yellow circles represent selected terpenoid-related VOCs; red lines indicate significant positive correlations (*r* > 0.9); blue lines indicate significant negative correlations (*r*< -0.9). The network represents statistical associations between gene expression and metabolite abundance and does not imply direct regulatory relationships.

These integrated analyses of P2–P4 revealed that many DEGs are involved in the terpenoid biosynthetic pathway. Positive correlations between genes and metabolites outnumbered negative ones, with 87 positive and 69 negative correlations identified (**Figure 8**). The network exhibited strong correlations between structural genes associated with terpenoid biosynthesis (*TPS04*, *TPS21*, *TPS22*, *MVK*, *ISPF*, *GGPPS*) and 20 selected terpenoid-related VOCs. *MVK*, an upstream gene in the terpenoid backbone biosynthesis pathway, was correlated with 12 terpenoid metabolites, suggesting a potential association between precursor-related metabolism and terpenoid VOC accumulation. *TPS22* was correlated with multiple terpenoid VOCs, including citronellol, myrcene, limonene, citral, geranyl acetate, eucalyptol, camphor, (E)-β-ocimene, with 16 correlated compounds in total, indicating that TPS22 is a promising candidate gene associated with terpenoid compositional variation. Carvyl acetate showed strong negative correlations with 26 genes, including *TPS04* (*r* = −0.93), *TPS21* (*r* = −0.92), *ISPF* (*r* = −0.93), and *GGPPS* (*r* = −0.92). These results indicate that complex positive and negative associations exist between the expression of terpenoid biosynthesis-related genes and terpenoid VOC accumulation. However, these associations should be interpreted as correlation-based candidate relationships rather than evidence of direct regulation.

## 4 Discussion

### 4.1 Floral scent characteristics of *Hesperis oreophila*

Floral scent is an important ornamental trait that enhances the economic value of flowering plants, and scent composition varies considerably among species. In the past, limited by detection techniques, researchers could only detect several dozen volatile metabolites from plant floral scents. Widely targeted metabolomics analysis, characterized by high throughput, high sensitivity, and broad coverage, has been widely recognized (Qi et al., 2024). In this study, based on HS-SPME-GC-MS technology, we systematically analyzed the volatile metabolome of *H. oreophila* at four key developmental stages (small bud stage, P1; large bud stage, P2; initial bloom stage, P3; full bloom stage, P4) for the first time, and detected a total of 1,211 volatile organic compounds (VOCs), encompassing 15 major categories including terpenoids, esters, heterocyclic compounds, ketones, aldehydes, and alcohols. In contrast, a classic study on the closely related species *Hesperis matronalis* identified only 33 volatile components from its flowers, with a relatively simple composition comprising only two major categories: terpenoids (mainly monoterpenes) and aromatics (benzene ring derivatives); complex aroma components such as esters, heterocyclic compounds, and long-chain aldehydes and ketones were not reported (Majetic et al., 2007). Comparison of the metabolite profiles of the two species revealed that putative aroma compounds such as α-pinene, β-pinene, myrcene, limonene, linalool, and eugenol are major scent constituents in both. However, *H. oreophila* exhibited pronounced species specificity and chemical diversity, producing terpenoids such as eucalyptol, camphor, (E)-β-ocimene, and nerolidol, which are key contributors to its rich sweet and woody fragrance, whereas these compounds have not been reported in *H. matronalis*. These results greatly enrich the candidate metabolite pool for floral scent in the genus *Hesperis* and update our understanding of floral scent.

Terpenoids represent the largest class of plant secondary metabolites and dominate the volatile profiles of many plants (Ramya et al., 2020; Liu et al., 2024). The primary floral scent compounds of *Rosa* spp (Koksal et al., 2015), *Jasminum* spp (Issa et al., 2020), *Lavandula* spp (Xiao et al., 2017), and *Paeonia* spp (Li et al., 2023). are all terpenoids. In the present study, terpenoids were also the most abundant class of metabolites. At the initial bloom and full bloom stages, they accounted for the highest proportion in terms of both compound number and abundance, reaching 166.25 μg·g^−1^ at full bloom and representing 35.8% of the total VOC content.

Although conventional OAV is widely used to evaluate the contribution of individual odorants to overall aroma, the present study employed rOAV because VOC abundances were obtained by semi-quantitative analysis rather than absolute quantification (Wang et al., 2020). Based on rOAV analysis, 108, 111, 187, and 206 metabolites with rOAV ≥ 1 were identified at the four floral developmental stages, respectively. Among them, the number of terpenoid compounds gradually increased, and they accounted for the highest proportion at both the initial bloom and full bloom stages. In addition, terpenoid compounds such as citronellol (Martin et al., 2017), myrcene (Yang et al., 2022), ocimene (Yeh et al., 2022), and linalool (Datta et al., 2024) were detected; these known terpenoids contribute to the floral scent of various species. In the present study, β-ionone, (+)-α-pinene, geraniol, limonene, and (E)-3,7-dimethyl-2,6-octadienal exhibited stable contents and high rOAVs (rOAV ≥ 70) throughout the flowering period and may therefore contribute to the floral scent profile of *H. oreophila*. However, these compounds should be regarded as putative aroma-contributing compounds because the rOAV analysis was based on literature-derived odor thresholds and semi-quantitative VOC contents. Definitive confirmation of their sensory contribution will require further validation using GC-olfactometry, sensory evaluation, aroma recombination/omission assays, or matrix-matched odor threshold determination. At P4, a relatively high proportion of DVOCs was associated with sweet, floral, herbal, and woody descriptors reported in the literature. Compared with P3, the representation of compounds associated with sweet and floral descriptors increased, whereas that of compounds associated with green descriptors decreased. Because these categories were assigned from published compound-level descriptions, this pattern represents a literature-based interpretation rather than a direct measurement of sensory intensity.

### 4.2 Expression patterns of genes related to floral scent in *Hesperis oreophila*

Transcriptome sequencing is a commonly used approach for studying plant metabolic networks and molecular regulatory mechanisms (Zhou et al., 2022). The discovery of key genes in volatile biosynthetic pathways has elucidated the molecular mechanisms regulating floral scent (Cai et al., 2016). To date, transcriptome sequencing has been widely applied to investigate the biosynthesis and regulation of floral scent compounds in plants such as *Dendrobium chrysotoxum* (Du et al., 2022), *Opisthopappus taihangensis* (Liu et al., 2022), and *Lonicera japonica* (Li et al., 2022). Nevertheless, the floral scent biosynthetic pathways in the genus *Hesperis* remain poorly understood. In this study, using floral organs of *H. oreophila* at flowering stages P2, P3 and P4, we performed transcriptome analysis to explore the pathways related to floral scent biosynthesis and characterized the expression patterns of candidate genes. A total of 107,689 representative unigenes were identified, of which 31,485 were differentially expressed. The gene expression differences gradually increased with flower development and were temporally associated with the dynamic divergence of volatile compounds, demonstrating that transcriptional changes were associated with VOC variation in floral scent formation in *H. oreophila*.

Plant floral scent is primarily produced through the biosynthesis of terpenoids, phenylpropanoids, and fatty acid derivatives (Liu et al., 2023). Among them, monoterpenes and sesquiterpenes constitute the major fraction of terpenoid volatile components and are the core compounds that shape the typical aroma of flowers (Qiao et al., 2021). The biosynthesis of terpenoids depends on the two precursors isopentenyl diphosphate (IPP) and dimethylallyl diphosphate (DMAPP), which are mainly synthesized via two independent pathways: the cytosolic mevalonate (MVA) pathway and the plastidial 2-C-methyl-D-erythritol 4-phosphate (MEP) pathway (Ramya et al., 2018). Comprehensive analysis based on sequence annotation and gene expression patterns across flowering stages revealed that genes encoding *DXS*, *DXR*, *HDR*, *TPS*, *GPPS*, and others are promising candidate genes regulating terpenoid biosynthesis (Huang et al., 2021). In this study, a total of 92 differentially expressed genes (DEGs) were annotated in the terpenoid backbone biosynthesis pathway, including 36 MVA pathway genes and 56 MEP pathway genes, covering the complete reaction cascade from upstream precursor synthesis to downstream catalysis by terpene synthases. Analysis of the expression dynamics of structural genes across different flowering stages revealed a typical asynchronous regulatory pattern between the two pathways: in the MVA pathway, 20 DEGs, including *ACAT*, *HMGS*, *HMGR*, *PMK*, *MVD*, and *FPPS*, reached their transcriptional peak at the large bud stage (P2) and were subsequently continuously downregulated at the initial bloom and full bloom stages. In contrast, 37 DEGs in the MEP pathway, including *DXS*, *DXR*, *ISPD*, *ISPF*, *ISPG*, *HDR*, *GPPS*, and *GGPPS*, were significantly upregulated at the initial bloom stage (P3). Notably, certain key genes within the pathways exhibited expression patterns opposite to the overall trend. This phenomenon suggests that different members of the same gene family may show stage-specific expression patterns and may be associated with the accumulation of different types of terpenoids during flower development. As an upstream enzyme-coding gene in the MEP pathway, *DXS* is generally considered important for precursor supply in terpenoid biosynthesis (Zhang et al., 2018); its expression in petals gradually increased from the bud stage to the full bloom stage, and its transcript abundance is closely associated with the monoterpene biosynthetic capacity of plants; however, in the present study, its role can only be inferred from transcript abundance and its temporal association with terpenoid accumulation. The two *DXS* genes identified in this study displayed pronounced stage-specific expression characteristics, being sequentially upregulated at the initial bloom and full bloom stages, respectively. This expression pattern is highly consistent with the continuous accumulation trend of terpenoid volatile compounds. These results suggest that different *DXS* family members may be transcriptionally associated with terpenoid accumulation at distinct developmental stages, but their specific biochemical functions require further validation. This expression pattern is consistent with findings reported for similar aromatic flower species. In studies of *Camellia* germplasm, the expression levels of *CaDXS2* and *CaDXS3* in the aromatic cultivars ‘Xiangtai’ and ‘Xiangfei’ were significantly higher than those in the non-aromatic cultivar ‘Chidan’, and both genes were upregulated at the full bloom stage, demonstrating that *DXS* is a core gene regulating monoterpene biosynthesis in *Camellia* (Chen et al., 2024). Research on Osmanthus fragrans has also revealed that the transcriptional rhythms of *OfDXS2* and *OfDHR1* are temporally associated with the diurnal emission dynamics of floral scent volatiles, further confirming that the temporal expression of key MEP pathway genes is an important mechanism regulating floral scent dynamics (Xu et al., 2016). Similarly, downstream terpene synthase (*TPS*) genes also exhibited asynchronous expression patterns: the majority of *TPS* members were predominantly expressed at the initial bloom stage, while a small number of genes showed high transcript levels at the large bud and full bloom stages, respectively. This phenomenon of temporally differentiated expression has been reported in *Chrysanthemum indicum* var. *aromaticum* and *Dendrobium chrysotoxum*, where studies have confirmed that the diverse biosynthesis of terpenoids at different flowering stages is significantly associated with the activation or repression of specific differentially expressed genes (Du et al., 2022; Zhu et al., 2022). Taken together, these pathway characteristics and gene expression patterns suggest that several terpenoid-related genes are temporally associated with changes in the type and abundance of floral scent volatiles. However, transcriptomic evidence alone is insufficient to determine their regulatory or enzymatic functions.

In addition, a total of 142 differentially expressed genes (DEGs) were annotated in the phenylpropanoid biosynthesis pathway, encoding six key enzymes including PAL, C4H, 4CL, CCR, CAD, and COMT. These genes exhibited pronounced stage-specific expression during flower development: 22 DEGs were highly expressed at the large bud stage, 73 DEGs at the initial bloom stage, and 43 DEGs at the full bloom stage. Similar to the temporal regulatory pattern observed in the terpenoid biosynthetic pathway, Similar to the temporal expression pattern observed in the terpenoid biosynthetic pathway, several core phenylpropanoid-related genes also showed elevated expression at the initial bloom stage, broadly coinciding with the expression patterns of MEP pathway and TPS genes.

Taken together, the gene expression characteristics of the terpenoid backbone biosynthesis and phenylpropanoid biosynthesis pathways revealed that the vast majority of DEGs involved in floral scent biosynthesis exhibited high transcript levels at the initial bloom stage, which was temporally associated with the significant increase in volatile metabolites detected by GC-MS at this stage. This indicates that the initial bloom stage represents a major developmental transition for floral scent formation in *H. oreophila*.

In this study, VOC profiling covered all four developmental stages, whereas RNA-seq was performed only for P2–P4. This design was based on the metabolomic evidence that P1 and P2, both representing bud stages, had relatively similar VOC profiles and clustered closely in PCA and hierarchical clustering analyses. By contrast, the P2–P3 transition exhibited the most pronounced metabolic shift, marked by a substantial increase in terpenoid accumulation and the onset of active scent formation. Thus, P2 was selected as the bud-stage transcriptomic baseline, while P3 and P4 represented the key scent-emission stages. However, the absence of P1 transcriptome data limits the direct inference of transcriptional changes from the small bud stage to later stages, and this consideration has been taken into account in the interpretation of the results.

### 4.3 Gene–metabolite association network related to terpenoid biosynthesis

Based on the correlation analysis between key structural genes in the terpenoid biosynthetic pathway and volatile metabolites, this study constructed a gene–metabolite association network centered on structural genes, providing a preliminary framework for screening candidate genes associated with terpenoid VOC accumulation in *H. oreophila*. Within this network, several genes from the MVA/MEP pathways and downstream terpene synthase (*TPS*) members all exhibited strong and significant correlations with multiple terpenoid VOCs, suggesting that these genes may be associated with stage-dependent terpenoid accumulation during flower development.

Among them, the upstream backbone synthesis gene *MVK* was significantly correlated with 12 terpenoid metabolites, suggesting a potential association between upstream precursor-related metabolism and downstream terpenoid VOC accumulation. The terpene synthase gene *TPS22* was highly correlated with 16 terpenoid VOCs, including citronellol, myrcene, limonene, and eucalyptol, suggesting that *TPS22* is a promising candidate gene associated with terpenoid compositional variation. Meanwhile, certain metabolites, such as carvyl acetate showed significant negative correlations with candidate genes such as *TPS04*, *TPS21*, *ISPF*, and *GGPPS*, indicating complex negative associations between terpenoid-related gene expression and metabolite accumulation. These associations may reflect coordinated or divergent accumulation patterns, but they should not be interpreted as evidence of inhibitory regulation without functional validation.

Similar to gene–metabolite association patterns reported in various aromatic plants, the expression patterns observed in the present study suggest that upstream pathway genes may be associated with precursor supply, while the temporally differentiated expression of different *TPS* members may be related to the accumulation of distinct terpenoid types. These results support the hypothesis that key structural genes in the terpenoid biosynthetic pathway are associated with the diversity and abundance of floral scent VOCs in *H. oreophila*. Nevertheless, the current evidence is based on transcript–metabolite correlations and should be considered exploratory rather than definitive proof of regulatory function.

Nevertheless, Pearson correlation analysis only reflects statistical co-variation between gene expression and metabolite abundance and cannot directly demonstrate causal regulation or enzymatic function. Therefore, *MVK*, *DXS*, *TPS21*, *TPS22*, and related genes should be considered candidate genes putatively associated with terpenoid VOC accumulation rather than confirmed functional regulators. Future studies combining recombinant enzyme assays, transient expression, gene silencing or overexpression, and substrate–product analysis, as applied in functional studies of floral *TPS* genes in other aromatic plants, will be required to verify the biochemical roles of these candidates (Abbas et al., 2020; Bao et al., 2023; Gao et al., 2018; Li et al., 2023).

## 5 Conclusion

In this study, VOC profiling was performed across four developmental stages of *Hesperis oreophila*, while transcriptome sequencing and integrated transcriptome–metabolome analysis were conducted for P2–P4, the major scent-transition stages, to characterize the dynamic accumulation patterns of floral scent compounds and the transcriptional patterns associated with VOC accumulation. We constructed, for the first time, a gene–metabolite association network for terpenoid scent biosynthesis in this species, thereby filling a gap in the molecular understanding of floral scent formation in the genus *Hesperis*. Our results demonstrate that terpenoids constituted the predominant VOC class at P3 and P4 and were considered potential contributors to the floral scent profile of *H. oreophila*, with their abundance increasing sharply from P2 to P3 and reaching a maximum at P4. The temporal accumulation of putative aroma-contributing compounds, including citronellol, myrcene, limonene, and (E)-β-ocimene, was associated with a developmental shift from a higher representation of compounds assigned to green descriptors at the bud stage toward a more diverse set of compounds assigned to sweet and related descriptors at full bloom. The initial bloom stage represents a transitional phase during which increased VOC accumulation occurs, with several terpenoid- and phenylpropanoid-related genes showing coordinated expression patterns at this stage. At the transcriptional level, MVA-, MEP-, and TPS-related genes showed distinct temporal expression patterns: the MVA pathway showed relatively high expression at the large bud stage, whereas several MEP pathway and downstream *TPS* genes were upregulated at the initial bloom stage, suggesting temporally distinct transcriptional patterns associated with terpenoid VOC accumulation. The gene–metabolite association network identified *MVK*, *DXS*, and *TPS22* as candidate genes correlated with terpenoid VOC accumulation and compositional variation. Among them, *TPS22* showed strong correlations with multiple characteristic terpenoids and may represent a promising candidate for future functional analysis of terpenoid compositional variation. However, because the current evidence is based on transcript abundance and metabolite correlation, these genes should be regarded as correlation-based candidates rather than confirmed functional regulators. The coexistence of expression divergence among homologous genes within the pathway and both positive and negative statistical associations further reflects the regulatory complexity of the terpenoid metabolic network. This study provides a theoretical foundation and candidate gene resources for the horticultural application and molecular breeding of *H. oreophila* and offers new perspectives for research on the regulation of secondary metabolism in wild Brassicaceae plants. Future work involving functional validation of core genes and identification of upstream regulatory factors will help refine the floral scent association network and support targeted trait improvement in aromatic flowers.

## Data Availability

The datasets generated in this study have been deposited in established repositories. The metabolomics dataset, including the converted GC–MS mzML files and associated metabolite annotation, abundance, sample metadata, and rOAV result files, has been deposited in MetaboLights under accession number: MTBLS15051. The quality-filtered paired-end RNA-sequencing reads have been deposited in the NCBI Sequence Read Archive under BioProject accession number: PRJNA1493792, with SRA accession number: SRR39600458–SRR39600466.
