# Supplementary material for: Floral scent profiling and candidate gene discovery in Hesperis oreophila
Source: Front Plant Sci. 2026 Aug 3;17:1922330. doi: 10.3389/fpls.2026.1922330 (PMC13478145; doi:10.3389/fpls.2026.1922330)
Supplement: Supplementary file 1 [file DataSheet1.docx]

Supplementary Material

# 1 Supplementary Figures and Tables

## 1.1 Supplementary Figures

**Supplementary Figure 1. OPLS-DA score plots for pairwise comparisons of *H. oreophila* at different flower developmental stages.** OPLS-DA score plots are shown for the six pairwise comparisons among P1 (small bud), P2 (large bud), P3 (initial bloom), and P4 (full bloom): P1 vs. P2, P1 vs. P3, P1 vs. P4, P2 vs. P3, P2 vs. P4, and P3 vs. P4. Each model contained one predictive component and one orthogonal component. Each point represents an individual biological replicate (*n* = 3 per stage), and different colors indicate different developmental stages. The horizontal axis represents the predictive score, whereas the vertical axis represents the orthogonal score. Separation between groups reflects differences in their overall VOC profiles.

**Supplementary Figure 2. S-plots derived from OPLS-DA models for pairwise comparisons of different flower developmental stages.** S-plots are shown for the six pairwise comparisons: P1 vs. P2, P1 vs. P3, P1 vs. P4, P2 vs. P3, P2 vs. P4, and P3 vs. P4. In each plot, the x-axis, *p*[1], represents the covariance or contribution of each VOC variable to model separation, whereas the y-axis, *p*(corr)[1], represents the corresponding correlation and reliability. Variables located toward the upper-right or lower-left extremes contributed relatively strongly to the separation between the two compared groups. VIP and S-plot information was used for supplementary interpretation of variable contributions rather than as a mandatory criterion for DVOC identification.

**Supplementary Figure 3. Permutation tests for evaluating the robustness of the OPLS-DA models.** Results of 200 permutation tests are shown for the six pairwise comparisons: P1 vs. P2, P1 vs. P3, P1 vs. P4, P2 vs. P3, P2 vs. P4, and P3 vs. P4. During permutation testing, sample-group labels were randomly reassigned and the OPLS-DA models were recalculated. The *R*² and *Q*² values of the original and label-permuted models are displayed in each panel. The permutation results supported that the separation observed in the original models was stronger than that expected from random reassignment of group labels, thereby supporting model robustness.

**Supplementary Figure 4.** **Phenylpropanoid biosynthesis pathway diagram.** Black bold font represented compounds. Red italicized font represented abbreviations of coding genes: *PAL*, phenylalanine ammonia-lyase; *C4H*, cinnamate 4-hydroxylase; *4CL*, 4-coumarate-CoA ligase; *CCR*, cinnamoyl-CoA reductase; *CAD*, cinnamyl alcohol dehydrogenase; *COMT*, caffeic acid O-methyltransferase.

## 1.2 Supplementary Tables

| Supplementary Table S1. Volatile metabolites with the top ten fold changes in each comparison group | | | |
| --- | --- | --- | --- |
| **Group** | **Compounds** | **Class I** | **Type** |
| P1 VS P2 | Benzeneacetic acid, methyl ester | Ester | up |
|  | Benzene, (1-methylethyl)- | Aromatics | up |
|  | Bicyclo[3.1.0]hex-2-ene, 4-methylene-1-(1-methylethyl)- | Terpenoids | up |
|  | Camphene | Terpenoids | up |
|  | .alpha.-Pinene | Terpenoids | up |
|  | (1R)-2,6,6-Trimethylbicyclo[3.1.1]hept-2-ene | Terpenoids | up |
|  | (1S)-2,6,6-Trimethylbicyclo[3.1.1]hept-2-ene | Terpenoids | up |
|  | Bicyclo[3.1.0]hex-2-ene, 2-methyl-5-(1-methylethyl)- | Terpenoids | up |
|  | Furan, 2-butyltetrahydro- | Heterocyclic compound | up |
|  | Myroxide | Terpenoids | up |
| P1 VS P3 | 2-Isobutyl-3-methylpyrazine | Heterocyclic compound | up |
|  | Benzofuran, 2,3-dihydro- | Heterocyclic compound | up |
|  | Phenol, 3-(1-methylethyl)- | Phenol | up |
|  | Benzene, (2,2-dimethoxyethyl)- | Ether | up |
|  | 1-Cyclohexene-1-carboxaldehyde, 2,6,6-trimethyl- | Terpenoids | up |
|  | Citronellol | Terpenoids | up |
|  | 6-Octen-1-ol, 3,7-dimethyl-, (R)- | Terpenoids | up |
|  | Amantadine | Amine | up |
|  | Methyleugenol | Phenol | up |
|  | Benzene, (1-methylethyl)- | Aromatics | up |
| P2 VS P3 | 2-Isobutyl-3-methylpyrazine | Heterocyclic compound | up |
|  | Ethanone, 1-(2-hydroxy-5-methylphenyl)- | Ketone | up |
|  | 2-Methoxy-4-vinylphenol | Phenol | up |
|  | Methyleugenol | Phenol | up |
|  | Amantadine | Amine | up |
|  | (1R,3aS,5aS,8aR)-1,3a,5a-Trimethyl-4-methylenedecahydrocyclopenta[c]pentalene | Terpenoids | up |
|  | Phenol, 3-(1-methylethyl)- | Phenol | up |
|  | Cyclopentanecarbonitrile, 3-(1-methylethylidene)- | Nitrogen compounds | up |
|  | Bicyclo[2.2.1]heptan-2-ol, 1,7,7-trimethyl-, formate, endo- | Ester | up |
|  | Isobornyl formate | Ester | up |
| P1 VS P4 | 2-Isobutyl-3-methylpyrazine | Heterocyclic compound | up |
|  | Methyleugenol | Phenol | up |
|  | Benzene, (1-methylethyl)- | Aromatics | up |
|  | Benzofuran, 2,3-dihydro- | Heterocyclic compound | up |
|  | Camphene | Terpenoids | up |
|  | Benzo[b]thiophene, 2-ethyl- | Heterocyclic compound | up |
|  | 2-Cyclohexen-1-ol, 3-methyl-6-(1-methylethyl)- | Terpenoids | up |
|  | Phenol, 3-(1-methylethyl)- | Phenol | up |
|  | .alpha.-Pinene | Terpenoids | up |
|  | (1R)-2,6,6-Trimethylbicyclo[3.1.1]hept-2-ene | Terpenoids | up |
| P2 VS P4 | Methyleugenol | Phenol | up |
|  | 2-Isobutyl-3-methylpyrazine | Heterocyclic compound | up |
|  | (1R,3aS,5aS,8aR)-1,3a,5a-Trimethyl-4-methylenedecahydrocyclopenta[c]pentalene | Terpenoids | up |
|  | Benzo[b]thiophene, 2-ethyl- | Heterocyclic compound | up |
|  | Phenol, 2,4-bis(1-methylethyl)- | Phenol | up |
|  | Phenol, 2,5-bis(1-methylethyl)- | Phenol | up |
|  | 2-Cyclohexen-1-ol, 3-methyl-6-(1-methylethyl)- | Terpenoids | up |
|  | Benzofuran, 4,5,6,7-tetrahydro-3,6-dimethyl- | Terpenoids | up |
|  | Ethanone, 1-(2-hydroxy-5-methylphenyl)- | Ketone | up |
|  | 2-Methoxy-4-vinylphenol | Phenol | up |
| P3 VS P4 | Acetic acid, phenyl ester | Ester | up |
|  | Menthyl acetate | Terpenoids | up |
|  | Benzene, (2-isothiocyanatoethyl)- | Ester | up |
|  | Benzene, 2-methoxy-1,3,5-trimethyl- | Ether | up |
|  | 2,6,10-Trimethyltridecane | Terpenoids | down |
|  | 2-Methoxy-3,5-dimethylpyrazine | Heterocyclic compound | down |
|  | 1,3-Dithiane, 2-methyl- | Heterocyclic compound | down |
|  | 4-Tridecanone | Ketone | down |
|  | o-Toluidine | Amine | down |
|  | Aniline, N-methyl- | Amine | down |

| **Supplementary Table S2.** Quality control statistics and comparative analysis | | | | | | | | |
| --- | --- | --- | --- | --- | --- | --- | --- | --- |
| **Sample** | **Group** | **Raw Reads** | **Clean Reads** | **Clean Base(G)** | **Error Rate(%)** | **Q20(%)** | **Q30(%)** | **GC Content(%)** |
| P2-1 | P2 | 47156708 | 41310002 | 6.2 | 0.02 | 95.7 | 88.2 | 44.93 |
| P2-2 | P2 | 58781428 | 51244488 | 7.69 | 0.02 | 95.75 | 88.31 | 44.54 |
| P2-3 | P2 | 44235172 | 39582864 | 5.94 | 0.02 | 95.67 | 88.07 | 44.8 |
| P3-1 | P3 | 57124068 | 51249964 | 7.69 | 0.02 | 95.82 | 88.52 | 45.38 |
| P3-2 | P3 | 49616288 | 45670298 | 6.85 | 0.02 | 95.68 | 88 | 45.08 |
| P3-3 | P3 | 50808852 | 45640010 | 6.85 | 0.02 | 95.99 | 88.8 | 44.57 |
| P4-1 | P4 | 45674538 | 40098056 | 6.01 | 0.02 | 95.91 | 88.57 | 44.67 |
| P4-2 | P4 | 51925600 | 46352110 | 6.95 | 0.02 | 95.89 | 88.7 | 44.74 |
| P4-3 | P4 | 51172030 | 46561662 | 6.98 | 0.02 | 95.92 | 88.75 | 44.9 |

| **Supplementary Table S3.** Gene annotation statistics | | |
| --- | --- | --- |
| **Database** | **Number of Genes** | **Percentage (%)** |
| KEGG | 67594 | 62.77 |
| Nr | 88378 | 82.07 |
| SwissProt | 68858 | 63.94 |
| TrEMBL | 88235 | 81.94 |
| KOG | 56537 | 52.5 |
| GO | 76904 | 71.41 |
| Pfam | 61025 | 56.67 |
| Annotated in at least one Database | 90228 | 83.79 |
| Total Unigenes | 107689 | 100 |

| **Supplementary Table S4.** Correspondence between metabolite IDs and compound names in the correlation network | | | |
| --- | --- | --- | --- |
| **Index** | **Compounds** | **Class I** | **Formula** |
| KMW0583*150 | trans-.beta.-Ionone | Terpenoids | C13H20O |
| WMW0023*011 | (1R)-2,6,6-Trimethylbicyclo[3.1.1]hept-2-ene | Terpenoids | C10H16 |
| KMW0459*283 | 2,6-Octadienal, 3,7-dimethyl-, (E)- | Terpenoids | C10H16O |
| KMW0460 | Geraniol | Terpenoids | C10H18O |
| KMW0217*017 | D-Limonene | Terpenoids | C10H16 |
| KMW0234*016 | p-Cymene | Terpenoids | C10H14 |
| KMW0434 | 1-Cyclohexene-1-carboxaldehyde, 2,6,6-trimethyl- | Terpenoids | C10H16O |
| KMW0199 | .beta.-Myrcene | Terpenoids | C10H16 |
| KMW0609 | Citronellyl butyrate | Ester | C14H26O2 |
| XMW0951*347 | 2-Cyclohexen-1-ol, 2-methyl-5-(1-methylethenyl)-, acetate | Ester | C12H18O2 |
| NMW0074*039 | Bicyclo[3.1.1]hept-2-ene-2-methanol, 6,6-dimethyl- | Terpenoids | C10H16O |
| KMW0218*089 | Eucalyptol | Terpenoids | C10H18O |
| KMW0291 | Linalool | Terpenoids | C10H18O |
| KMW0446 | Citral | Terpenoids | C10H16O |
| NMW0055 | 6-Octenal, 3,7-dimethyl-, (R)- | Terpenoids | C10H18O |
| D163 | Camphor | Terpenoids | C10H14O2 |
| D186*282 | trans-Carveol | Terpenoids | C10H16O |
| QWM0003 | 2H-Pyran, 3,6-dihydro-4-methyl-2-(2-methyl-1-propenyl)- | Terpenoids | C10H16O |
| KMW0530*352 | Geranyl acetate | Terpenoids | C12H20O2 |
| WMW0040*154 | trans-.beta.-Ocimene | Terpenoids | C10H16 |

| **Supplementary Table S5.** Primers for quantitative real-time PCR | | |
| --- | --- | --- |
| **Gene** | **Primer** | **Primer sequence** |
| *Elongation factor-1a(EF1a)* | Forward primer | ATGGTGATGCTGGTATGG |
|  | Reverse primer | TCCTTCTTGTCAACACTCTT |
| *TPS-CIN* | Forward primer | TCTAGCCAATGATCTCGCAACT |
|  | Reverse primer | TCCTCCGAAGCACCTGTCT |
| *TPS14* | Forward primer | TCGAGACAAGAAGGGTGGATTT |
|  | Reverse primer | GCTCAGAGGCTTCGTACAATTC |
| *POLX* | Forward primer | TCCCAAGCAGAGCATACGAAAT |
|  | Reverse primer | GATACGCATCGAGGTGGAGAAT |
| *DXS* | Forward primer | GAGAGCGAGCACGATTCCTT |
|  | Reverse primer | CTGTCATGGCACCATCACCTAT |
| *TPS02* | Forward primer | GTGTTGAAGGAAGAGGTGAGGA |
|  | Reverse primer | TCGTAGTGGTGATGGTGAATGT |
| *ISPG* | Forward primer | TGCTTGTGGCTGAGATGTATGT |
|  | Reverse primer | TTGTGTCACCTAATCCGTCCTG |
| *KTI2* | Forward primer | AAGCAGGAGAAGAAGCAGGAG |
|  | Reverse primer | CAAGCCGAGTAGTGGAAGTAGT |
| *TPS21* | Forward primer | AAGGTCAGGTTGTGGCTTCC |
|  | Reverse primer | GCGAATGTCACGGCTATCAAG |
| *CYP82G* | Forward primer | CTCTAACCGCCTCCGCAAAT |
|  | Reverse primer | GGTTCCGAGCATCCACAATCT |
| *IGMT4* | Forward primer | TCTTCCAACCAAACCCACCAA |
|  | Reverse primer | ATTCAACCAGACGGCACTTCA |
| *PER71* | Forward primer | GCTTCAGGAACAGGCTATACGA |
|  | Reverse primer | CGCTTCCAGTGTCCATATCCA |
| *PAL1* | Forward primer | CTATGCTATGCGGAGAGGTGAA |
|  | Reverse primer | CGAGATTGCTGCCACTTGTC |
| *PER42* | Forward primer | TGTCCGCAAGAGAAGGCATC |
|  | Reverse primer | TACTCTCGTTGTGGTCAGGAAG |
| *BBE2* | Forward primer | CATCGGTGGTGGCTCTGTT |
|  | Reverse primer | CTGTGTGGCGTTATCGTTGTTT |
| *HSP70-6* | Forward primer | ACGAGTTCTTGCGGCTTGT |
|  | Reverse primer | CTGTCGGAACCTGGAGTAACC |
| *FBA4* | Forward primer | GTGTTCTGGCTGGAGATGTGA |
|  | Reverse primer | GGCAGTGTTGTGTTCCTTGG |

| **Supplementary Table S6.** Pearson correlation analysis between qRT-PCR and RNA-seq expression trends for the 16 validated DEGs | | | |
| --- | --- | --- | --- |
| **RNA-Seq** | **QPCR** | **Correlation** | ***P*-value** |
| *TPS-CIN* | *TPS-CIN* | 0.996184332 | 0.055631222 |
| *TPS14* | *TPS14* | 0.999456679 | 0.020986653 |
| *POLX* | *POLX* | 0.999529206 | 0.019535637 |
| *DXS* | *DXS* | 0.995429739 | 0.060887925 |
| *TPS02* | *TPS02* | 0.989883747 | 0.090629954 |
| *ISPG* | *ISPG* | 0.999965034 | 0.005323759 |
| *KTI2* | *KTI2* | 0.987669417 | 0.100076981 |
| *TPS21* | *TPS21* | 0.993357552 | 0.073417549 |
| *CYP82G* | *CYP82G* | 0.999232016 | 0.024951634 |
| *IGMT4* | *IGMT4* | 0.999608701 | 0.017809995 |
| *PER71* | *PER71* | 0.997013143 | 0.049216472 |
| *PAL1* | *PAL1* | 0.999639711 | 0.017089677 |
| *PER42* | *PER42* | 0.999591093 | 0.018206328 |
| *BBE2* | *BBE2* | 0.99013481 | 0.089496394 |
| *HSP70-6* | *HSP70-6* | 0.999999914 | 0.000264226 |
| *FBA4* | *FBA4* | 0.999687082 | 0.015926548 |

| **Supplementary Table S7.** Differentially accumulated volatile metabolites in pairwise comparisons across four flower developmental stages of Hesperis oreophila | | | | |
| --- | --- | --- | --- | --- |
| **Group number** | **Group name** | **All sig diff** | **Up regulated** | **Down regulated** |
| 1 | P1VSP2 | 196 | 180 | 16 |
| 2 | P1VSP3 | 621 | 592 | 29 |
| 3 | P1VSP4 | 703 | 668 | 35 |
| 4 | P2VSP3 | 571 | 548 | 23 |
| 5 | P2VSP4 | 708 | 682 | 26 |
| 6 | P3VSP4 | 219 | 181 | 38 |
